# Using Combined Diagnostic Test Results to Hindcast Trends of Infection from Cross-Sectional Data

**DOI:** 10.1371/journal.pcbi.1004901

**Published:** 2016-07-06

**Authors:** Gustaf Rydevik, Giles T. Innocent, Glenn Marion, Ross S. Davidson, Piran C. L. White, Charalambos Billinis, Paul Barrow, Peter P. C. Mertens, Dolores Gavier-Widén, Michael R. Hutchings

**Affiliations:** 1 Biomathematics and Statistics Scotland (BIOSS), Edinburgh, United Kingdom; 2 SRUC, Edinburgh, United Kingdom; 3 Environment Department, University of York, York, United Kingdom; 4 Laboratory of Microbiology and Parasitology, Faculty of Veterinary Medicine, University of Thessaly, Karditsa, Greece; 5 Department of Biomedicine, Institute for Research and Technology of Thessaly, Larissa, Greece; 6 School of Veterinary Medicine and Science, University of Nottingham, Nottingham, United Kingdom; 7 The Vector-Borne Viral Diseases Programme, The Pirbright Institute, Surrey, United Kingdom; 8 National Veterinary Institute (SVA), Uppsala, Sweden; University of Georgia, UNITED STATES

## Abstract

Infectious disease surveillance is key to limiting the consequences from infectious pathogens and maintaining animal and public health. Following the detection of a disease outbreak, a response in proportion to the severity of the outbreak is required. It is thus critical to obtain accurate information concerning the origin of the outbreak and its forward trajectory. However, there is often a lack of situational awareness that may lead to over- or under-reaction. There is a widening range of tests available for detecting pathogens, with typically different temporal characteristics, e.g. in terms of when peak test response occurs relative to time of exposure. We have developed a statistical framework that combines response level data from multiple diagnostic tests and is able to ‘hindcast’ (infer the historical trend of) an infectious disease epidemic. Assuming diagnostic test data from a cross-sectional sample of individuals infected with a pathogen during an outbreak, we use a Bayesian Markov Chain Monte Carlo (MCMC) approach to estimate time of exposure, and the overall epidemic trend in the population prior to the time of sampling. We evaluate the performance of this statistical framework on simulated data from epidemic trend curves and show that we can recover the parameter values of those trends. We also apply the framework to epidemic trend curves taken from two historical outbreaks: a bluetongue outbreak in cattle, and a whooping cough outbreak in humans. Together, these results show that hindcasting can estimate the time since infection for individuals and provide accurate estimates of epidemic trends, and can be used to distinguish whether an outbreak is increasing or past its peak. We conclude that if temporal characteristics of diagnostics are known, it is possible to recover epidemic trends of both human and animal pathogens from cross-sectional data collected at a single point in time.

## Author Summary

We have developed a Bayesian approach that can estimate the historic trend of incidence from cross-sectional samples, without relying on ongoing surveillance. This could be used to evaluate changing disease trends, or to inform outbreak responses. We combine two or more diagnostic tests to estimate the time since infection for the individual, and the historic incidence trend in the population as a whole. We evaluate this procedure by applying it to simulated data from synthetic epidemics. Further, we evaluate its real-world applicability by applying it to two scenarios modelled after the UK 2007 bluetongue epidemic, and a small outbreak of whooping cough in Wisconsin, USA. We were able to recover the epidemic trends under a range of conditions using sample sizes of 30–100 individuals. In the scenarios modelled after real-world epidemics, the hindcasted epidemic curves would have provided valuable information about the distribution of infections. The described approach is generic, and applicable to a wide range of human, livestock and wildlife diseases. It can estimate trends in settings for which this is not possible using current methods, including for diseases or regions lacking in surveillance; recover the pattern of spread during the initial “silent” phase once an outbreak is detected; and can be used track emerging infections. Being able to estimate the past trends of diseases from single cross-sectional studies has far-reaching consequences for the design and practice of disease surveillance in all contexts.

## Introduction

Infectious disease surveillance is the first line of detection and defence against infectious pathogens and therefore crucial to maintaining animal and public health. However, the current state of disease surveillance has been characterised as deficient in terms of both coverage and reporting speed for both humans [1] and animals [2,3]. The challenge is to use the data generated by this often sparse and biased surveillance to decide on an appropriate response to disease outbreaks. This is dependent on the extent of *situational awareness*, which can be defined as “Knowledge and understanding of the current situation which promotes timely, relevant, and accurate assessment … in order to facilitate decision making.” (taken from [4], p 171). Such situational awareness is necessary in order to balance the social and economic consequences of the adopted control strategy with the social and economic risks posed by the outbreak [5].

Limited situational awareness can have substantial negative impact. In the case of the pandemic H1N1 flu in 2009, early analyses mistakenly assumed that the epidemic had been only recently introduced, causing substantial overestimates of the basic reproduction ratio [6] and case fatality rates [7] that suggested a far greater risk to human life than was actually the case, leading to a more resource-intensive response than was necessary [8]. The more complex settings typical of livestock and particularly wildlife systems tend to result in the available surveillance data being sparser still for animal diseases [9].

Adding missing information on the time of exposure of detected cases would allow for a better awareness of the early development of an epidemic and would help inform evaluations of the potential risks posed by an outbreak, leading to a more proportionate response than would be the case when waiting for the epidemic trends to be revealed by subsequent real-time monitoring. In the current study, we introduce a novel statistical approach to infer the timing of exposure events for individuals by combining knowledge of the dynamic characteristics of multiple diagnostic tests. This approach could be integrated into any model of a disease epidemic to replace missing information on case exposure times. In this paper, we demonstrate its usefulness by recovering population-level trends of exposure from cross-sectional data collected from a single point in time. Here we refer to the process of recovering such trends as “hindcasting”, following terminology established in other papers [10–12] for reconstructing historical trends from currently available data.

Disease surveillance has been described [13] as improving the situational awareness in relation to a disease outbreak on three levels: Perception, Comprehension, and Projection. Perception refers to the collection of data that allows us to monitor disease; Comprehension to extracting information from this raw data that places the current disease situation in a context that allows us to understand its characteristics; and Projection to statistical models as well as more holistic approaches that aim to describe what is likely to happen in the future. Research focused on improving the collection of surveillance data [14–16], on risk-based surveillance [17,18], or the extensive literature focusing on the early detection of statistical deviations in surveillance data to outbreaks [19–21], can be seen as improving the Perception stage. Approaches such as phylodynamics contribute to the Comprehension stage by modelling the genetic change of the pathogen, e.g. using this to estimate the epidemiological parameters governing an outbreak such as the recent Ebola outbreak [22–24]. Models that use current information to predict the future [25–27] instead focus on improving the situational awareness at the Projection stage. From this perspective, hindcasting contributes to the Comprehension stage by leveraging quantitative diagnostic test results (using the statistical methods described in this paper) to add a temporal dimension to data for which the times of exposure of cases are missing, thus improving the understanding of unfolding epidemics.

Several papers have recovered limited historical characteristics of epidemics from cross-sectional data using a single diagnostic test, e.g. an antibody test. For example, Giorgi et al. estimated the time of the start of an HIV outbreak under assumptions of exponential growth of viral load [28]. Others have exploited information on diagnostic test kinetics, i.e., the pattern of diagnostic test values during the course of infection, to estimate average incidence rates. Examples include the use of antibody test kinetics to estimate sero-incidence rates for influenza [29], *Salmonella* in cattle [30] and *Salmonella* in humans [31]. One challenge in these kinds of studies is that the relationship between the magnitude of signals from diagnostic tests and time since exposure is usually not monotonic; they tend to increase and then decrease. This means that the inverse problem of estimating time since exposure given a test value is non-unique, and although this can be framed as a statistical problem the resulting inference is highly uncertain [28,32], limiting what can be estimated from test data. However, there are often several diagnostic tests available that target different aspects of the multi-faceted dynamic interaction between host and pathogen, and thus exhibit different test kinetics [33]. That is, the profile of test responses, as a function of time since exposure, will differ depending on the underlying diagnostic used and the immune-pathogenesis of the disease. Thus, in principle we can generate a unique signal for a given time since exposure by combining results of diagnostic tests that respond on different time scales. Here, we exploit this fact to develop a more robust statistical approach for analysing cross-sectional field data from multiple diagnostic tests. To do so we make use of empirical infection models that characterise test kinetics to infer the time since exposure for each individual. While there is considerable uncertainty in the estimated exposure time for each individual, the combined estimates from multiple individuals can be used to describe the overall population-level distribution of infection times and estimate the shape of the overall epidemic trend with a high level of confidence.

**Fig 1:**
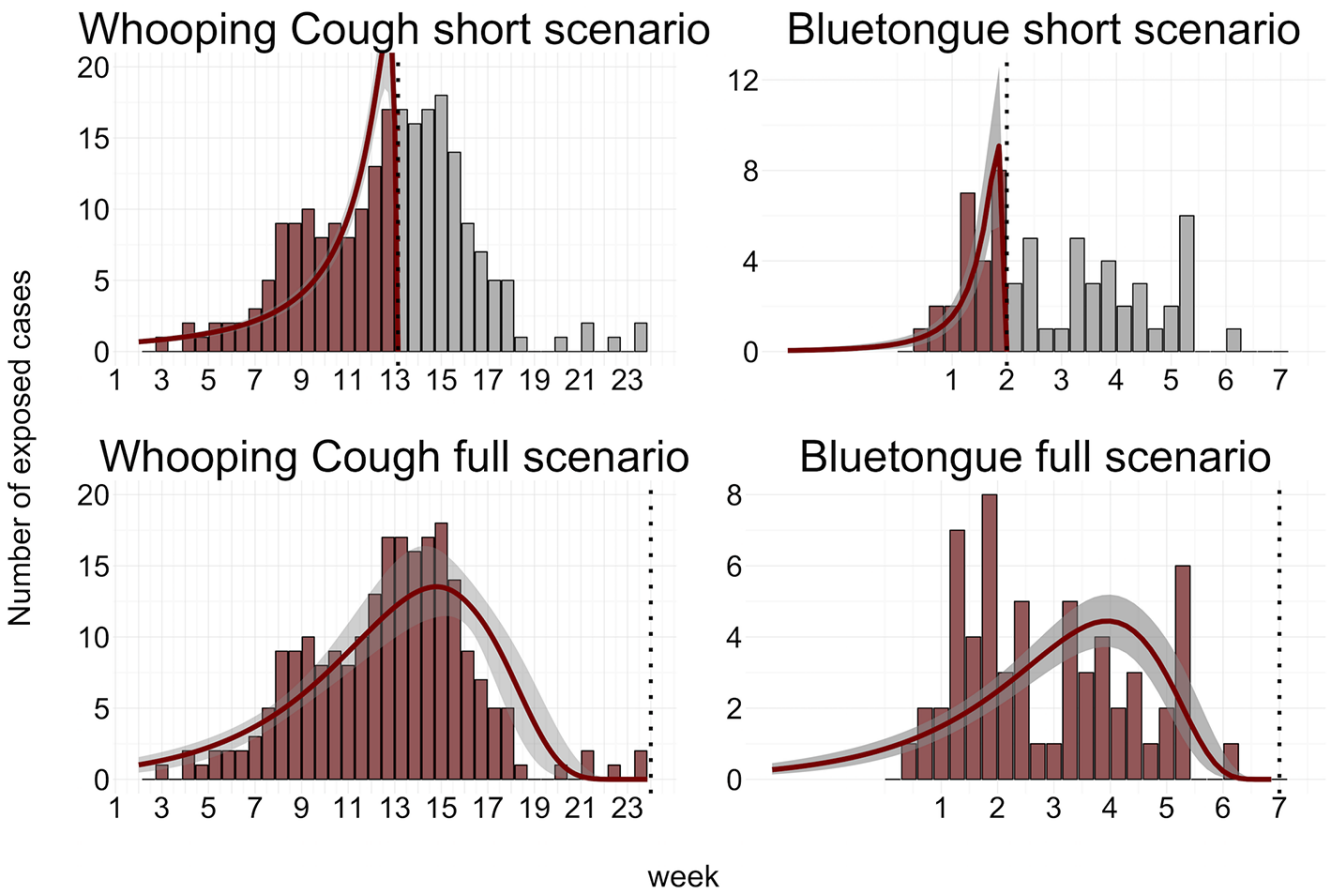
Outbreak scenarios together with estimated epidemic curves. Top left: Testing 100 whooping cough cases at week 35 of a 2003 Wisconsin outbreak. Top Right: Testing 100 bluetongue cases at week 7 of the 2007 UK outbreak. Bottom left: Testing 25 cases at week 25 of the Wisconsin outbreak. Bottom right: Testing 30 cases at week 3 of the 2007 UK outbreak. In all scenarios, cases were sampled from the full population of cases shown in the outbreak data of Fig 1 that had been exposed before the time of testing. Vertical dashed lines indicate time of cross-sectional sample. Red bars indicate cases included in the sampling frame for testing, grey bars indicate cases not included (note that hindcasting is designed to estimate historic, not future, trends). Red lines indicate the mean posterior hindcast trend based on the cross-sectional test data. The grey transparent regions around the trends indicate the 95% posterior credible interval.

A detailed description of the hindcasting framework and implementation of the evaluation scenarios can be found in the methods section. We demonstrate the hindcasting of epidemic trends by applying the framework developed here to case studies of real outbreaks of two contrasting diseases, whooping cough in humans and bluetongue in cattle (see Fig 1). For each disease, we investigate two scenarios representing detection during either the increasing or the decreasing phase of the epidemic. We conclude that when combined with knowledge of the temporal characteristics of two (or more) appropriate diagnostic tests, our methods allow historical epidemic trends to be recovered from cross-sectional sample data. Moreover, for the example diseases considered suitable diagnostic tests and data describing their temporal characteristics already exist.

## Results

We evaluated the hindcasting framework by applying it to simulated data sets, and comparing the recovered trend with the known underlying distribution. We first generated collections of exposure times from lognormal probability distributions with different sets of parameters. Using published test kinetics for whooping cough, we then generated diagnostic test data based on these infection times and an assumed cross-sectional sampling time. The hindcasting framework was applied to these generated test data, and the estimated posterior distribution for the epidemic trends was compared to the known simulated epidemic trend. In order to explore the real-world applicability of this approach, we also simulated diagnostic test data using published test kinetics and published distributions of case reporting times, from an outbreak of bluetongue in the UK in 2007, and from an outbreak of whooping cough in Wisconsin in 2003.

### Recovering the parameters of lognormal epidemic trends

We generate exposure times from four different lognormal distributions, each representing a different epidemic scenario as follows:

$$Epi1∼logN\left(\text{log}\left(\mu \right)=\text{log}\left(2\right),\text{log}\left(\sigma \right)=\text{log}\left(\sqrt{5}\right)\right)$$

$$Epi2∼logN\left(\text{log}\left(\mu \right)=\text{log}\left(4\right),\text{log}\left(\sigma \right)=\text{log}\left(\sqrt{5}\right)\right)$$

$$Epi3∼logN\left(\text{log}\left(\mu \right)=\text{log}\left(20\right),\text{log}\left(\sigma \right)=\text{log}\left(\sqrt{2}\right)\right)$$

$$Epi4∼logN\left(\text{log}\left(\mu \right)=\text{log}\left(50\right),\text{log}\left(\sigma \right)=\text{log}\left(\sqrt{2}\right)\right)$$

where the above notation means that the exposure times in each epidemic are drawn from the corresponding log-normal distribution. These represents epidemics peaking 2, 4, 20, and 50 days before the time of sampling, with the relative standard deviation chosen to provide more and more gradual increasing trends.

We found that we could reliably recover the epidemic trends when using sample sizes of 30 or more, and with levels of test variability less than 1.5, and that the estimated trend showed better fit when the peak was less recent than if it had just occurred (likely due to a difficulty in resolving very rapid dynamics relative to diagnostic test characteristics).

**Fig 2:**
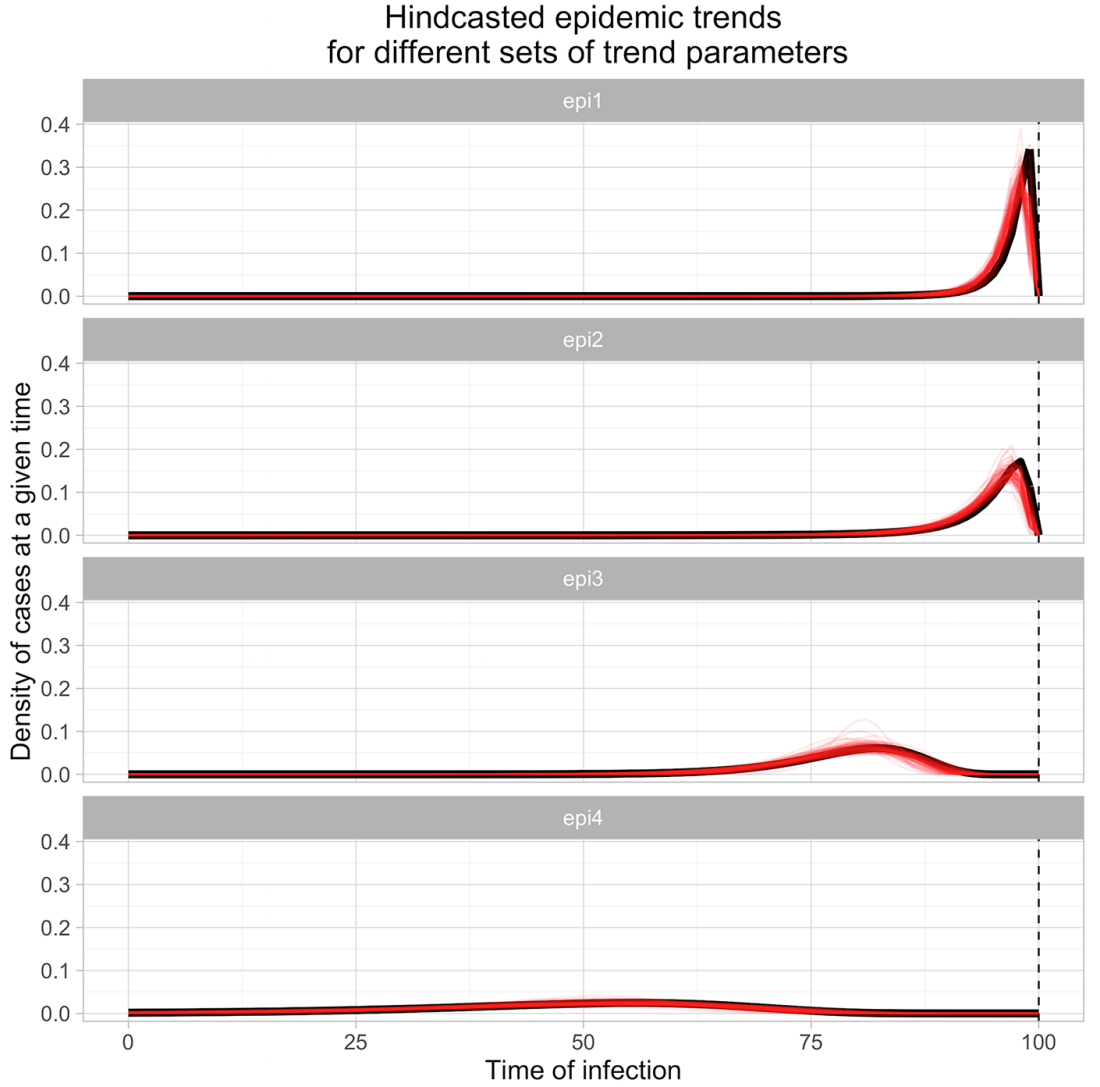
Hindcasted epidemic trends compared to the true lognormal trends. Each row shows 80 hindcasted posterior mean trends (each estimated trend shown in thin red lines), estimated from between 30 and 100 test results, with exposure times generated using one of four different lognormal epidemic trends (see main text; true trend shown in black), and assuming a lognormal measurement error of 1.3 for the diagnostic tests. Vertical dashed line indicate the time of the cross-sectional sampling.

Fig 2 shows the hindcasted trends for 320 simulations that were conducted with a sample size of between 30 and 100, with a test variability of 1.3, evenly split across the four parameterizations. As can be seen, these trends all manage to adequately capture the timing and duration of the true epidemic, with a clear separation between the estimates for different sets of true parameter values.

Turning to summary statistics of the epidemic fit across these sets of posterior mean trends, the median R^2^ (and root mean squared error of prediction—RMSEP) for the Epi1 parameterisation was 0.71, with a 95% inter-quantile range (IQR) of 0.19–0.97 (RMSEP of 0.054[0.021–0.109]), a median of 0.85 with a 95% IQR of 0.48–0.99 (RMSEP of 0.019[0.005–0.041]) for the Epi2 parameterisation, a median of 0.96 with a 95%IQR of 0.64–0.998 (RMSEP of 0.005[0.01–0.020]) for the Epi3 parameterisation, and a median R^2^ of 0.97 with a 95% IQR of 0.69–0.999 (RMSEP 0.002[0–0.007]) for the Epi4 parameterisation.

**Fig 3:**
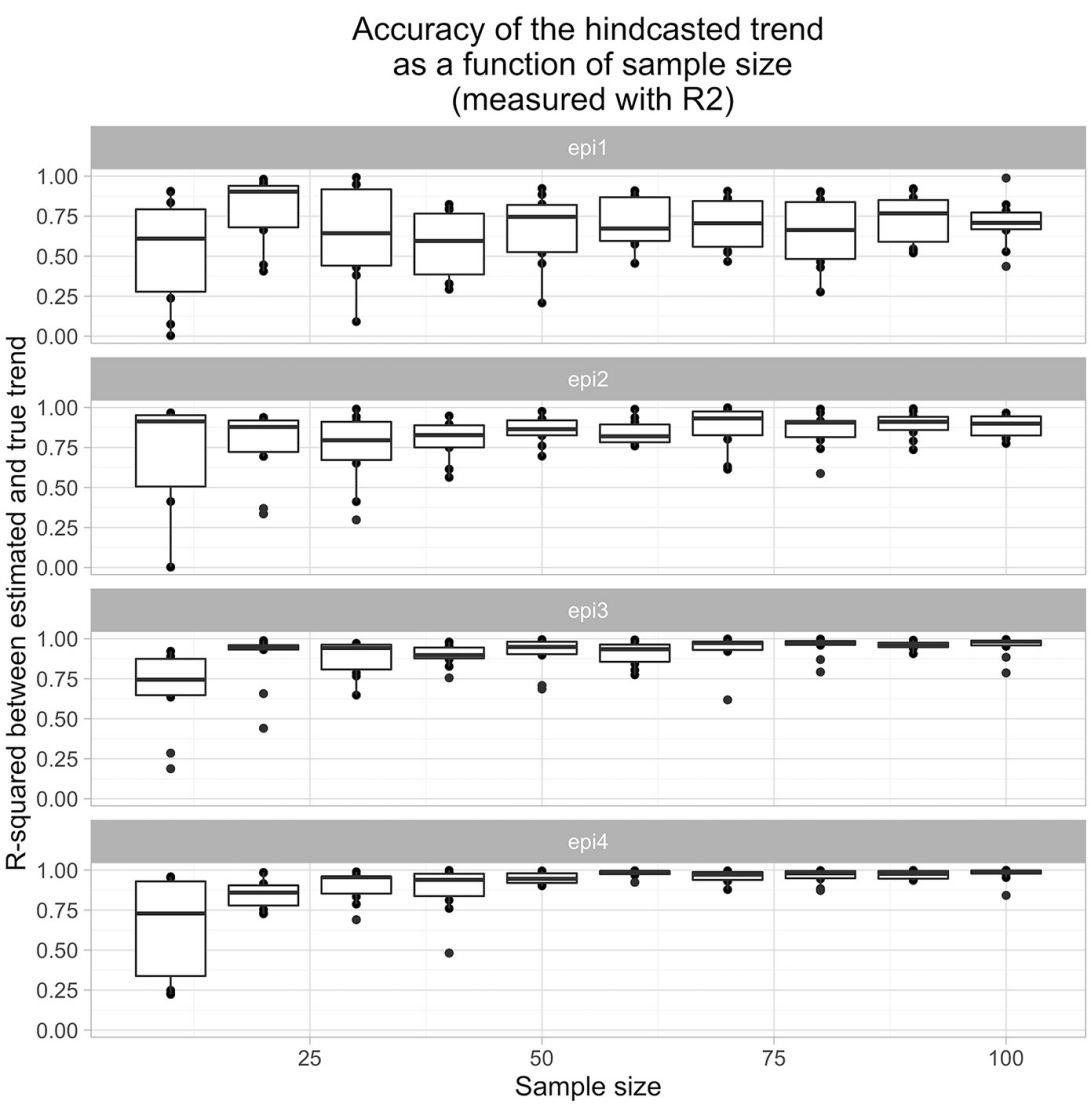
Relationship between prediction accuracy and sample size, as measured with R^2^. Each boxplot represents the results of applying the hindcasting framework to ten different data sets generated with the same set of parameters.

Fig 3 shows the relationship between sample size and estimation performance. As can be seen, increasing the sample size improved the performance as measured with R^2^ for all of the parameterizations except Epi1 (fitting Epi1 was limited by the time resolution of the diagnostic test kinetics used). The posterior credible intervals for the parameters of the epidemic also shrunk in width, as would be expected. The performance when hindcasting using sample sizes of 10 was not very reliable; however, for sample sizes of 30 or more, the recovered trends reliably represented the true trend, with R^2^ values of 0.75 or more for all parameterizations except Epi1.

### Robustness of the hindcasting framework

In order to evaluate the robustness of the hindcasting framework, we explored a range of testing errors from 1.1 up to 2.0 (multiplicative standard deviation). We found that that the width of the credible intervals increased moderately with increasing variability, but that the recovered parameters exhibited similar levels of bias regardless of the level of test variability. This was true even for testing errors of as high as 2.0, far beyond the reported variability of the examined diagnostic tests for bluetongue and whooping cough. (See S1 Text for full plots regarding the relationship between test variability and performance.)

Finally, we investigated the effect of violating the assumption of conditional independence of antibody and nucleic acid tests. Changing the amount of correlation from 0 up to 1 in 0.25-unit intervals showed no detectable difference in the results, whether measured with R^2^, RMSEP, or parameter estimates. (See S1 Text for related figures.)

### Case studies

**Fig 4:**
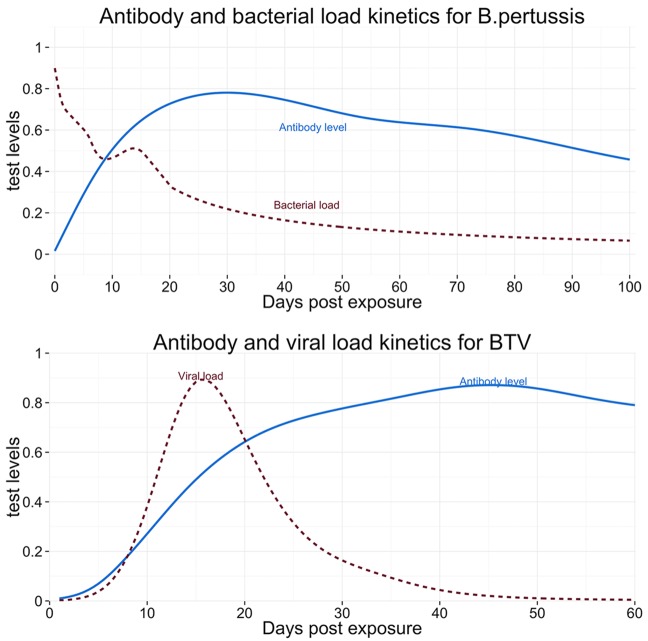
Graphs of the kinetics of diagnostic test kinetics used in the paper. Top: Diagnostic test kinetics for whooping cough, with an antibody test (solid line) and a test measuring bacterial load (dashed line). Bottom: Diagnostic test kinetics for bluetongue, with an antibody test (solid line), and a test measuring viral load (dashed line). The graph is showing idealised test kinetics, based on published data for whooping cough [34,35] and bluetongue [36] tests(see methods for details).

We also applied the hindcasting framework to two case studies based on a recorded outbreak of whooping cough in humans, and a bluetongue outbreak in cattle (see Fig 1). See the methods section for details. For each outbreak we simulated two scenarios, firstly where a random subset of all individuals exposed thus far was sampled and tested at a single time, midway through the outbreak (increasing epidemic trend/early detection), and in a second scenario where a random subset of all exposed cases were sampled and tested at a time point at the end of the outbreak (decreasing epidemic trend/late detection). We assumed that no information about the time since exposure was available, nor any other information about the epidemic trend. Based on published temporal characteristics of real diagnostics, test results were then simulated for these samples (see Methods and Fig 4). For each disease (whooping cough and bluetongue) and each scenario (increasing and decreasing outbreaks) the hindcasting framework was applied to the corresponding test results to assess performance in recovering early increasing phases and late decreasing phases of outbreaks.

The results show that the recovered epidemic trends provided a representative picture for both increasing and decreasing scenarios, in both whooping cough and bluetongue outbreaks (Fig 1). For the increasing whooping cough epidemic, when assuming a sample of all 122 cases that had occurred between the start of the epidemic up to week 25, the R^2^ between underlying case counts (smoothed by a 7-day moving average) and the estimated epidemic trends was 0.74, with a 95% confidence interval of [0.69–0.78]. The corresponding RMSEP was 0.0017[0.0015–0.0018] When sampling 230 cases from the full whooping cough epidemic up until week 36, after it had declined, the curve fit was somewhat better, with R^2^ of 0.82[0.68–0.94] (RMSEP 0.0013[0.0008–0.0017]).

The results from hindcasting the bluetongue outbreak indicated that when assuming that a sample of the 26 animals had occurred during the increasing phase, the fitted curve was nearly perfect, with an R^2^ of 0.9[0.86–0.92] (RMSEP 0.0019[0.0018–0.002]). However, for the corresponding decreasing scenario, assuming a sample of the 61 animal cases that had occurred up to week seven, the hindcast trend could not fully capture the erratic nature of the underlying case count data, as indicated by R^2^ values of 0.21[0.15–0.27]). The trend did indicate an elevated incidence over the stretch of time when the majority of cases occurred, thus capturing the approximate time that had elapsed between the start of the epidemic and the time of sampling. This aspect is also captured by the RMSEP, which is more sensitive to shifts in locations, and less sensitive to upward- and downward trends, and which slightly improved to 0.0016[0.0015–0.0016].

When reducing the sample size, the hindcasting technique was still able to recover both increasing and decreasing phase for the whooping cough scenarios. The good fit was maintained with sample sizes as low as 20 individuals, with R^2^ values of 0.77[0.27–0.83] (RMSEP 0.0064[0.0055–0.0124]), for the increasing and 0.67[0.09–0.86] (RMSEP 0.0038[0.0022–0.0069]) for the decreasing scenario. The performance was also maintained for the increasing bluetongue scenario, also assuming 20 samples, with an R^2^ of 0.91[0.87–0.93]) (RMSEP 0.042[0.034–0.051]). However, the full bluetongue scenario performed substantially worse with the reduced sample size with an R^2^ of 0.12[0.07–0.36] (RMSEP 0.014[0.013–0.015]).

### Benefit of two tests

**Fig 5:**
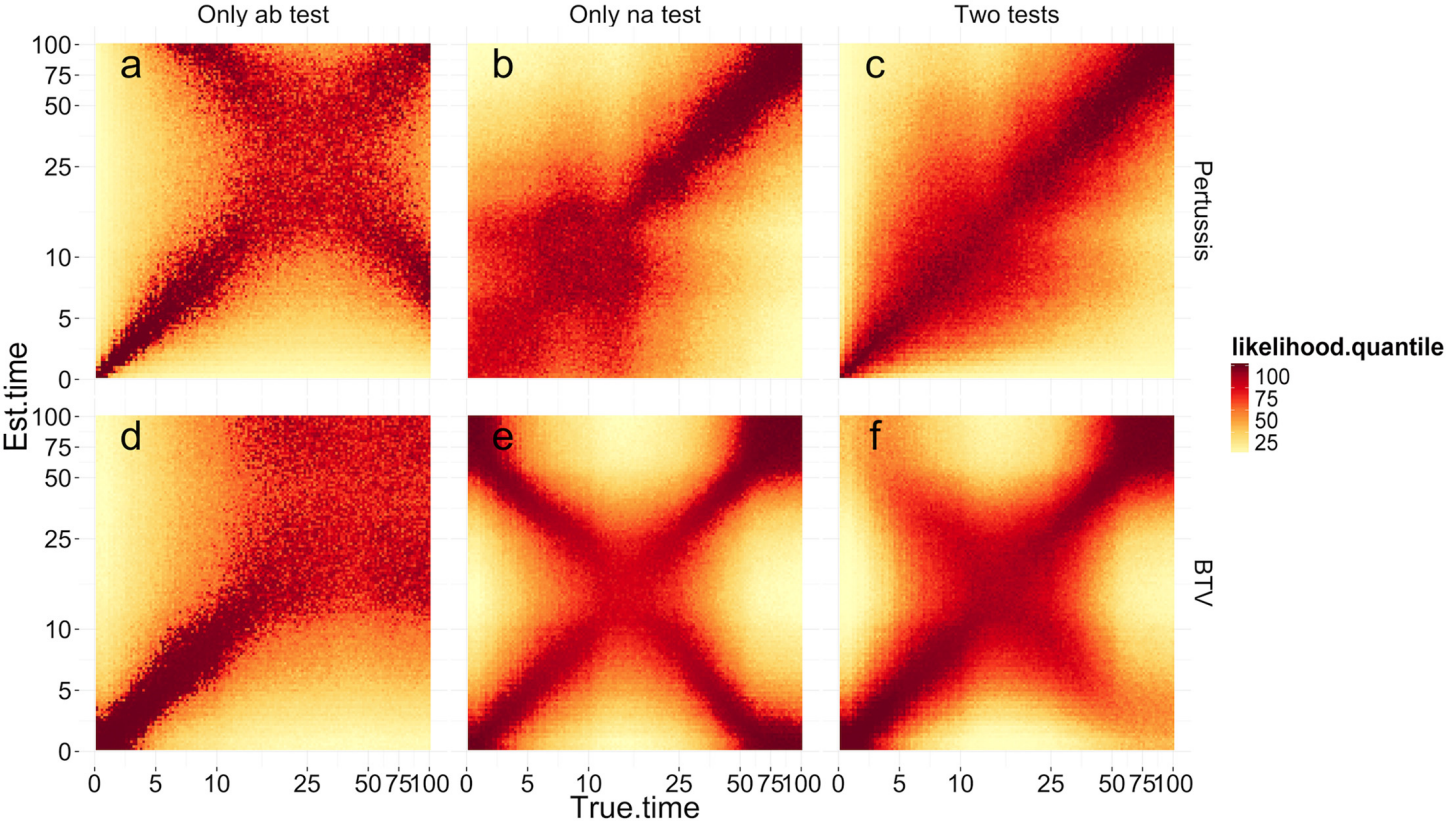
Log likelihood of inferred times of exposure as a function of true time since exposure. The log likelihood values plotted are conditional on test data generated assuming that the individual was exposed to whooping cough (top row) or bluetongue (bottom row) at the true time. Both the X and Y axes are on a log scale. Each pixel represents the value of the likelihood at a time of exposure given by the Y axis, given 10 test results, generated assuming a time since exposure given by the X axis. The colour of the pixel indicates the likelihood for an estimated time, given the sample data, with dark red being most likely, and pale yellow being least likely. A clear, dark red diagonal indicates that the time since exposure is easily recoverable, while a more diffuse diagonal indicates higher levels of uncertainty (see the results section for details). The first column shows results based only on data from the antibody test relevant to the disease in question, the middle column results based on an appropriate nucleic acid test, and the right hand column shows the results based on both tests. (See text for details.)

We further investigated how the hindcasting framework would be affected by different combinations of test kinetics. Fig 5 shows the mean likelihood surface of true vs. posterior times of exposure when using antibody-based tests, nucleic-based tests, or a combination, for the whooping cough and bluetongue exemplar diseases. For the hindcasting framework the ideal combination of diagnostic tests would have a likelihood surface with a single narrow diagonal representing a maximum likelihood coinciding with the true exposure time given observed data. A likelihood surface with a more diffuse diagonal implies a wider posterior distribution. A likelihood surface where there is an “X” of high-likelihood regions implies that the true exposure times are not uniquely identifiable.

Each pixel represents the average likelihood of 10 observations from the distribution of test measurements at a time since exposure given by the X axis, calculated at a time of exposure given by the Y axis. Areas in dark red indicate regions of higher likelihood. The times shown are times since exposure, with low numbers indicating more recent exposure, where the test response is changing rapidly. Looking at Fig 5a, during the first 20 days the probable exposure times (red pixels), given the data, are centred on the diagonal (i.e. the true exposure times) with a narrow band of high-probability red pixels. For times since exposure of greater than 20 days, when the kinetics of the antibody test are developing at a slower pace, the diagonal of red pixels becomes more diffuse, indicating a greater variation around the true times since exposure. Furthermore, we can see that there are two different diagonals crossing at 25 days. This corresponds to the peak of the diagnostic response curve, with the two diagonals indicating the possibility that a given test result could have been the result of testing an individual during either the increasing or the decreasing phase of the response curve. Estimation of the time since exposure is more precise when the true time since exposure corresponds to phases where the response is changing rapidly, and is more difficult to infer when the test response levels out (Fig 5b and 5d). For diagnostic tests with a peaking response, estimating the time of exposure can be precise but not unique, with two different regions of probable exposure times for a given test response (Fig 5e).

To further evaluate the gain from utilizing two different tests, we ran simulations against the four parameterizations of a lognormal epidemic mentioned above, using two diagnostic tests and starting with respectively whooping cough and bluetongue test kinetics. The kinetics were then modified to be increasingly similar to each other (full details and results in S1 Text). We found that for the bluetongue scenarios, the level of similarity of the tests did not seem to noticeably affect the accuracy (as measured with R^2^/RMSEP) of the estimated trends, while for the whooping cough scenarios when recovering Epi3 and Epi4 scenarios, performance degraded gradually, with a very low R^2^ when using two identical tests. bluetongue test kinetics, the MCMC sampler converged well for the unmodified test configuration (as measured using Gelmans R statistic), but the convergence behaviour then degraded as the tests became increasingly similar. It completely failed to converge in the limit when the two tests were identical (either identical NA tests, or identical antibody based tests). For the whooping cough scenarios the sampler converged for all combinations of diagnostic tests, no matter how similar.

## Discussion

We have shown how to recover epidemic trends of both human and animal pathogens from cross-sectional data collected at a single point in time. We were able to recover this temporal information using a novel statistical framework which combines paired diagnostic test measurements made on collected samples with known temporal kinetics of diagnostics test measurements over the course of infection.

The inferential framework introduced here allow us to extract rich temporal information from collected diagnostic samples. Here we focused on purely cross-sectional samples, but the methods are applicable to longitudinal data and data sets combing both longitudinal and cross-sectional samples. We were able to estimate the trends of both increasing epidemics and decreasing epidemics, as well as estimate the approximate pace of increase or decrease. Such information would improve situational awareness during outbreaks, enabling appropriate management decisions to be implemented immediately when an outbreak has been detected, without the need to observe its subsequent spread to estimate the trend.

The implementation of the framework used in this paper combines surveillance data with information on the test kinetics using a simplified model. For example, individual variation in the test response is modelled as variation around a common mean test curve, rather than as variation in the shape of the curve itself. Variations in the two tests are considered independent, and the error distribution is assumed to be log normal. This limits the pattern and range of variation our model can capture, but facilitates model specification and estimation. We also assume that the test variability is known. While this is partly owing to technical limitations (models with unknown variance parameters tended to converge to degenerate solutions by maximising the variance), it is a realistic assumption since the reliance on test kinetics require that the diagnostic test has been studied in depth. More detailed modelling of the individual and population level processes (including the effect of various covariates such as age or gender) in order to tailor the model to a particular disease is entirely consistent with the statistical framework introduced and would increase the real-world validity and predictive power beyond what has been demonstrated here.

The current implementation of the framework does not include a sampling process component, and so the generated posterior distribution does not currently take sampling uncertainty into account. If the samples are randomly drawn from a larger population of infected individuals, the estimated trend will be an unbiased estimator of the wider population trend. A potential avenue for future research would be to integrate hindcasting into a wider framework describing the sampling process in detail; such an approach might also allow for simultaneously estimating potential sampling bias.

We make use of the lognormal distribution as a parsimonious parameterization of the epidemic trend. This is suitable for epidemics where a single peak is expected, allowing fast model fitting whilst capturing the time span and general direction of the trend. The trade-off is that more complex aspects of trends in the epidemic are omitted. A second limitation is that the lognormal distribution requires the trend to decline to zero after any peak. Should either of these limitations pose a problem, more complex parameterizations of the epidemic trend—with multiple peaks and stages, or even compartmental SEIR-type models—could be used, though such models are likely to come at substantially higher computational cost.

The hindcasting framework introduced here estimates epidemic trends by combining observed data with information on how test responses develop after exposure. Woolhouse and Matthews [37] give an extensive overview of studies that incorporate different data sources to recover the underlying dynamics of disease spread [38,39], and argue that the future of disease analysis lies in models taking account of a wider range of inputs, such as diagnostic test performance, disease pathogenesis, or transmission mechanics, in addition to regular surveillance data. Our methodology improves on earlier studies incorporating test kinetics [29–31] in three ways: by incorporating information from more than one diagnostic test; by considering their joint kinetic pattern; and by modelling non-constant incidence. It could be further extended to model other aspects of the disease system such as population demography, contact networks, or the spatial distribution of cases.

The hindcasting approach make use of knowledge of the within-host development of test markers. Phylodynamics, on the other hand, leverages information about how the genetics of the pathogen change as it spreads through the population to estimate between-host transmission events, and use this to e.g. reconstruct the transmission network of outbreaks [40] and to inform future control measures and forecasts of outbreak trajectories such as the 2015 Ebola outbreak [41], and the 2009 H1N1 influenza outbreak [42]. However, phylodynamics requires sequenced samples of genetic material, and that the pathogen of interest is mutating quickly enough that the dynamics of the epidemic can be resolved. In contrast, the hindcasting approach relies on test kinetics and measures within-host times since infection. Recent papers [43,44] discuss ways to integrate epidemiological and genetic information when modelling disease epidemics; given the complementary nature of phylodynamics and hindcasting, a natural future step would be to combine the two sources of information into a single framework.

Since hindcasting exploits knowledge of the host-pathogen interaction, it relies on previously conducted longitudinal studies of such interactions, and requires that the test response after initial pathogen exposure has been described. Our results demonstrate one of the many ways in which experimental infection studies can provide substantial additional benefits to disease control and research. Currently, only a fraction of pathogen tests have published information on how time since exposure affects test response; this has limited which pathogens we could usefully simulate. Similarly, data sets of infectious diseases often only record whether a test has been positive or not. Presentation of the underlying continuous test response is rare—and it is rarer still to find such results for paired diagnostic tests.

It is hoped that the method introduced here can give some further motivation to record continuous test responses from more than one diagnostic test, and that it can also serve as an argument for conducting further studies on test kinetics. The results regarding the impact of combining diagnostic tests indicate that combining diagnostic tests increases the robustness of the hindcasting procedure. The bluetongue scenarios exhibited severely degraded convergence behaviour with two identical tests, while the whooping cough scenarios converged, but produced posterior estimates that did not match the true trend.

Further research is required to characterise how combinations of tests interact to affect hindcasting. In general, however, the diagnostic tests used in disease surveillance should be chosen to complement each other. By combining early responding tests with later responders, it becomes possible to create a joint test signature that combines the best features of both tests. In terms of the method presented here, the best combination of tests is likely that which provides a unique and precise signature along the timeline of infection for an individual.

The hindcasting framework described here makes use of diagnostic test information, that has up until now been under-utilized, to improve situational awareness during an outbreak.

This approach could also be used to improve the detection of new outbreaks by extracting more information from existing surveillance data and thus make outbreak detection algorithms [20,21] more sensitive. It could similarly be used to provide additional sources of information when estimating epidemiological parameters and trends, and thus improve the accuracy of forecasting models.

We have described a new framework for hindcasting the temporal patterns of epidemics, using two example host-pathogen systems and the pairing of antibody tests with pathogen load. The framework demonstrates the potential to utilise the information inherent in the increasing variety of diagnostic tests. We were able to estimate both increasing and declining epidemic trends under the assumption that all individuals were being tested at a single point in time, implying its usefulness for cross-sectional surveillance data as well as in less restrictive settings. Recovering temporal incidence trends using multiple tests on cross-sectional field data has the potential to be of considerable value, and is a key determinant of introducing proportionate responses to ongoing disease outbreaks.

## Methods

### Statistical framework

Our method assumes test data *y*_*ik*_ from multiple disease diagnostics indexed by *k* = 1, …, *K* on individuals *i* = 1, …, *N*. We assume that each individual is tested at some time *t*_*i*_, after having been exposed to the pathogen at some earlier time *e*_*i*_. We further assume that these individuals are chosen in an unbiased, random manner from a larger population. Each diagnostic test is assumed to return a value in the form of a continuous ‘level’, which might, for example, be the highest dilution at which antibodies are detected in a serological test. Without loss of generality we assume that these levels are scaled to the unit interval [0,1].

Initial exposure to a pathogen is the start of a complex dynamical process within the host. We conceptualize such internal host-pathogen interactions as a multivariate process that depends on the time since initial exposure. Each diagnostic test is assumed to target the state of a different component of this process so that each test *k* carried out at time *t*_*i*_ on individual *i* can be modelled as a latent variable *l*_*ik*_(*t*_*i*_, *e*_*i*_) = *l*_*ik*_(*d*_*i*_), with each test having differing but correlated response patterns over the time since initial exposure *d*_*i*_ = *t*_*i*_−*e*_*i*_. We model these latent variables using results from experimental infection studies for a given host-pathogen system, where the length of time since initial exposure *d*_*i*_ is known.

The known data, across all individuals in the sample, comprises a set of test results denoted by ***Y*** = {*y*_*ik*_} with sampling times ***T*** = {*t*_*i*_}. Our aim is to infer the unknown set of exposure times ***E*** = {*e*_*i*_}, using information on the behaviour of the latent processes ***L*** = ***L***(***T*, *E***) = {*l*_*ik*_(*t*_*i*_, *e*_*i*_)} generating the test results. Note that when describing these sets the limits of each index *k* = 1, …, *K* and *i* = 1, …, *N* are implicit.

Under our statistical model we assume that the sampling times ***T*** are precisely known whereas the quantities ***Y*, *L*** and ***E*** are assumed to be subject to uncertainty and variation. There are thus three components to the statistical model: a latent process model *P*(***L***|***T***, ***E***, *θ*_*L*_) describing uncertainty and variation in the host-pathogen interaction process within the host in terms of the time since initial exposure; a testing or observation model *P*(***Y***|***L***, *θ*_*Y*_) describing the distribution of results from tests carried out on the hosts conditional on the internal latent process; and an epidemic trend model *P*(***E***|***T***, *θ*_*e*_), describing the historical development of the epidemic in terms of the distribution of exposure times in the sampled host population, at the time of sampling. We discuss specific implementations of each of these components in the examples described below.

Combining the three parts of the model, we write the full data likelihood given an observed data set {***Y***, ***T***}as

$$P\left(Y,E,L|T,\theta \right)=P\left(Y|L,\theta_{Y}\right)P(L|T,E,\theta_{L})P(E|T,\theta_{E}),$$

where *θ* = {*θ*_*Y*_, *θ*_*L*_, *θ*_*E*_}. Thus the likelihood combines models for testing with those for within and between host pathogen interactions.

According to Bayes’ theorem, the so-called posterior distribution for the unknown parameters is proportional to the data likelihood and prior P(θ). We can express this relationship for the parameters of interest, the latent process ***L***, the exposure times ***E*** and the parameters *θ*, given the observed test data ***Y*** and sampling times ***T***, by the equation

$$P\left(L,E,\theta |Y,T\right)=\frac{P\left(Y,E,L|T,\theta \right)\text{P}\left(\theta \right)}{P\left(Y,T\right)}$$

Within the Bayesian framework all inference is based on the posterior. The prior P(θ) can result from previous measurements or expert opinion, and represents knowledge about the values of parameters before we see the data used in the likelihood.

In what follows, we will make the simplifying assumption that the latent process ***L*** is modelled by a known deterministic function of ***T*** and ***E***, and represents the expected value of the test results given the times since exposure. This means that the term *P*(***L***|***T***, ***E***, *θ*_***L***_) drops out of the likelihood which then simplifies to *P*(***Y***, ***E|T***, *θ*) = *P*(***Y***|***L***(***T***, ***E)***, *θ*_*Y*_)*P*(***E***|*θ*_***E***_), and the posterior becomes

$$P\left(E,\theta |Y,T\right)=\;\frac{P\left(Y,E|T,\theta \right)P\left(\theta \right)}{P\left(Y,T\right)}$$

Note that under this notation any parameters defining the deterministic latent process ***L***(***T***, ***E***) = {*l*_*nk*_(*t*_*n*_, *e*_*n*_)} are suppressed since they are not inferred i.e. *θ* = {*θ*_*Y*_, *θ*_*E*_}.

In both cases above the normalisation factor *P*(***Y***, ***T***) is typically unknown and computationally expensive to calculate. However, standard Markov Chain Monte Carlo (MCMC) methods circumvent this problem and are able to generate samples from the posterior even though the normalisation is unknown. The results presented in this paper are generated from an MCMC sampler implemented with a Metropolis-Hastings algorithm in JAGS [45] using Gibbs sampling [46].

### Case studies

Whooping cough is a human disease caused by the bacteria *Bordetella pertussis*, causing prolonged spasmodic coughing. Despite widespread vaccination coverage there has been a resurgence of cases in several countries. In the Netherlands there has been a steady increase in the incidence since 1996; and in California, USA, in 2011, there was a widespread outbreak with 9000 cases and ten deaths [47]. The reasons for such resurgence is currently a matter of scientific debate; some hypotheses include antigenic drift of the bacterium [48,49], asymptomatic transmission of *B*. *pertussis* by vaccinated individuals [50], or the resurgence being the consequence of changing vaccines and vaccination schedules [51]. Here we make use of data describing a county-wide outbreak of whooping cough primarily among adolescents and adults in Fond du Lac County, Wisconsin, USA in 2003–2004, [52]. After an early cluster of cases in a high school in early May 2003, there was a large outbreak of whooping cough throughout the county starting from October. After some time, this outbreak was contained, and the final cases occurred in February 2004. The upper part of Fig 1 shows interpolated case counts per 48-hour period over the duration of the outbreak.

Bluetongue virus (BTV) is a midge-borne virus that can infect ruminants such as sheep, cattle, deer, and camelids, causing bluetongue disease with symptoms such as internal haemorrhages, swelling of the tongue, lesions in the mouth, and in some species death (most notably in naïve sheep and white-tailed deer). Bluetongue infections can have severe economic consequences for livestock farming, both due to loss of productivity, and because of the severe control measures needed to prevent spread [53]. In 2006, bluetongue emerged throughout northern Europe, with recorded outbreaks in the Netherlands, Belgium, Germany, and Luxembourg. In 2007, the UK had its first recorded outbreak [54]. The first infections occurred sometime in early August 2007 [54] when midges introduced the pathogen to the British Isles, but the first case was not detected until September. The lower part of Fig 1 shows the case count per day, with numbers interpolated from the published weekly data [54].

In order to assess our methodology, we consider two scenarios for each pathogen outbreak. In the “increasing” scenarios we assume the epidemic is recognised early and explore test results from samples taken at a time early on in the outbreak (when the outbreak is increasing, see e.g. Fig 1). In contrast in the “decreasing” scenarios we use test results assumed to be obtained from individuals exposed during the entire outbreak, with samples collected at a relatively late stage in the outbreak (i.e. when it is in decline). The goal was to see how well hindcasting could distinguish between increasing scenarios and scenarios where the epidemic is in decline. We were also interested to see if it was possible to estimate the approximate time span of the epidemics.

#### Implementation of the whooping cough scenarios

We based our whooping cough data set on the case count curve of the 2003 Wisconsin whooping cough outbreak. We used published bi-weekly case counts, and interpolated these using a LOESS [55] approach to generate estimated 48-hour case counts.

We investigated two hypothetical scenarios for when the outbreak could have been first detected and cases tested. We simulated one scenario where we assumed that a random subset was selected from all cases that had occurred between the start of the epidemic and 25 weeks after the first observed case. The selected cases were assumed to be sampled and samples tested at the 25 week time point. At this time point, the first wave had passed, and the second sharp increase in incidence had been going on for about a month. 126 cases had been reported by this time in the actual outbreak. The second scenario assumed that cases were selected from the 230 cases from the full whooping cough epidemic up until week 36, and that samples were taken and tested at that time. This time point marks the end of the epidemic, with no later cases reported.

#### Implementation of the bluetongue scenarios

As with whooping cough, we assumed two different hypothetical scenarios for when the outbreak was noticed and animals tested: one assuming that a random subset of the 26 exposed animals up week two were sampled and tested at the end of the second week, and the other assuming that a random subset of the 61 animals exposed by the end of week seven were sampled and samples tested at this time (Fig 2).

### Generation of simulated test results

The results of diagnostic testing are characterised in terms of an underlying mean trend and a model which accounts for variation around this reflected measurement error, and within and between individual variability in test response.

Given simulated times of exposure, we then simulated test results, based on the elapsed time between the time of exposure in the outbreak and the assumed time of sampling, using published kinetics of real-time PCR analysis and quantitative ELISA for *B*. *pertussis* [34,56], to inform a latent process *P*(***L*|*T***, ***E***, *θ*_*L*_). Specifically these were the kinetics of ELISA IgG *B*. *pertussis* antitoxin [35] for antibody test response *ab*(*d*)as a function of time since exposure *d*, and real-time PCR measurement of persistence over time of *B*. *pertussis* DNA in nasopharyngeal secretions [34] (see Fig 2) for the pathogen load *DNA*(*d*). As noted earlier formally, we defined the deterministic function ***L***(*d*_*i*_) = (*DNA*(*d*_*i*_), *ab*(*d*_*i*_)) by fitting interpolated curves to the published data on DNA and antibody levels using LOESS [55].

The distribution *P***(***Y*_*i*_|***L***(*d*_*i*_)) of test measurements was modelled as a lognormal distribution conditional on the state of the latent process: let ***y***_***i***_ = (*y*_*NA*_, *y*_*ab*_)_*i*_ represent a bivariate measurement of nucleic acid and antibody levels on individual *i*, and define the distribution $P\left(Y_{i}|L\left(d_{i}\right)\right)=\;lN\left(L\left(d_{i}\right),\Sigma^{2}\right))$, where *Σ*^2^ is a diagonal covariance matrix, reflecting the assumption of no correlation between test results when conditioned on the time since exposure. The variance for each test (i.e. the diagonal elements of *Σ*^2^) was assumed to be known. Antibodies as well as levels of pathogens in a host often follow log-normal distributions, as has been rigorously argued [57]; the suitability of using the lognormal distribution for modelling a wide range of biological phenomena has also been described more recently [58].

We modelled the test behaviour based on published data [36], and assumed lognormal distributions for the epidemic trend, as well as for the variance of the diagnostic tests (test kinetics shown in Fig 4). Specifically, we based the behaviour of the latent process *P*(***L***|***T***,***E***, *θ*_*L*_) on a study of experimental infection of European red deer with BTV serotype 1 and 8 that described the dynamics of BTV serotype 1 viral load (*vl*) as measured with RT-PCR, and antibody levels (*ab*) as measured with ELISA. As above, we define the latent process describing antibody concentration and viral load as a deterministic bivariate function of the duration *d* elapsed since exposure as ***L*** = {***l***(*d*_*i*_)} ≡ (*vl*(*d*_*i*_), *ab*(*d*_*i*_)), which does not vary between individuals. We estimate ***L*** by fitting smooth and interpolated curves to the experimental study data on viral load and antibody levels independently and take the values of these curves at each exposure time *d* to define the values of the deterministic functions, *vl*(*d*), *ab*(*d*). A smoothing spline algorithm [55] was used as a nonparametric fitting method. Conditional on the time since exposure, the observed test values ***y***_*i*_ = (*y*_*vl*_, *y*_*ab*_)_*i*_ were modelled as a bivariate log-normal distribution with mean equal to the deterministic latent process = {***l***(*d*_*i*_)} = (*vl*(*d*_*i*_), *ab*(*d*_*i*_)). For individual *i*, this can be formally written as $P\left(y_{i}|l\left(d_{i}\right)\right)=lN\left(l\left(d_{i}\right),\Sigma^{2}\right)$, where lN indicates a bivariate lognormal probability function, and *Σ*^2^ is the covariance matrix. We assumed that the variation in observed antibody levels and viral loads to be independent so that the covariance matrix Σ^2^ is diagonal, with variance components $\sigma_{1}^{2},\;\sigma_{2}^{2}$. The variance for each test (i.e. the diagonal elements of *Σ*^2^) was assumed known.

### Modelling the epidemic trend

The third and final part of the model, the distribution of times since exposure *P*(***E***|***T***, *θ*_*e*_), was modelled as a lognormal distribution $P\left(E|T,\;\theta_{e}=\left\{\mu ,\sigma \right\}\right)=lN\left(\mu ,\;\sigma \right)$.

In this case, we exploit the ability of the lognormal to model extreme skewness to capture both increasing and decreasing epidemics using only two parameters. Note that the implementation of the lognormal distribution as an epidemic trend must be conducted in such a way as to allow the sampler to jump between tails of the distribution for the individual times since infection. Details for how to do this can also be found in the supplementary information.

### Choice of priors

We followed the recommendations of Gelman et al. [59] and used vague priors for the parameters. Such priors incorporate information about what parameter values are nonsensical in a given problem setting, but without using any previously collected data. The means for the lognormal distribution describing the epidemic trends were themselves given lognormal priors. These priors were set to indicate the timescale of relevance for the epidemics in question. This translated to setting the prior means for the for the increasing whooping cough to 100 days, and prior means for the decreasing whooping cough scenario to 200 days. The corresponding values for bluetongue were 10 days and 100 days, respectively. The standard deviations for the prior distributions were chosen as log(10) corresponding to 99% confidence intervals of (mean/100,mean*100). This standard deviation was chosen to model that any peak time more than a factor 100 different from the time scale of interest was nonsensical.

The standard deviation of the lognormal distributions for the epidemic trend was given a vague prior parametrized as a folded, non-standardized t-distribution with five degrees of freedom, a standard deviation of log(100), and a mean of 0, indicating in the spirit of vague priors that a spread of on the order of more than 100 days in the past was not sensible.

In the process of developing this work, we also explored the use of uninformative priors with nearly flat distributions, such as the standard gamma distribution, and uniform priors with very wide support; however, these were found to lead to very slow mixing and a high rate of convergence failure of the MCMC algorithm. Changing the specific values of the priors did not influence the posterior estimates noticeably. See the supplementary information for MCMC traceplots, details of convergence evaluation and sensitivity analysis of the priors.

## Supporting Information

S1 Text Extended simulation results, details on data generation and implementation and convergence evaluation of the MCMC sampler. (DOCX)
