# Supplementary material for: Using Combined Diagnostic Test Results to Hindcast Trends of Infection from Cross-Sectional Data
Source: PLoS Comput Biol. 2016 Jul 6;12(7):e1004901. doi: 10.1371/journal.pcbi.1004901 (PMC4934910; doi:10.1371/journal.pcbi.1004901)
Supplement: S1 Text — (DOCX) [file pcbi.1004901.s001.docx]

Supporting information

**Using combined diagnostic test results to hindcast**

**trends of infection from cross-sectional data**

Gustaf Rydevik, Giles Innocent, Glenn Marion, Ross Davidson, Dolores Gavier-Widen, Paul Burrow, Pete Mertens, Charalambos Billinis, Mike Hutchings

**Extended results of the simulation study**

**Robustness against diagnostic test measurement error**

In order to thoroughly test the robustness of the hindcasting framework, we ran a set of simulations with four values for the diagnostic test error: 1.1, 1.4, 1.7, and 2.0 (these numbers are all referring to the multiplicative (or exponent) of the standard deviation of the lognormal error distribution, centred at the mean test level given the test kinetic and time since exposure). We used four different sample sizes (25, 50, 75, and 100 samples), the four sets of parameterizations of the epidemic trend described in the main text, and generated 10 different data sets for each unique combination of these three parameters.

Figures S1 and S2 shows the impact of the amount of test error used when generating data on the performance of the hindcasting framework as measured with R^2^ and RMSEP. Judging by the plot with RMSEP (Fig. S2) the hindcasting framework showed uniformly poor performance when applied to Epi1, similar to what was seen in the main simulation results (see main text). For the other three parameterizations, however, the performance was maintained at similar levels for all levels of test error as long as the sample size was 50 or more. Fig S1 tells a similar story, though indicate a certain degradation of performance for a testing error of 2.0, which might be because R^2^ is more sensitive to shifts in location between compared curves than RMSEP.

Figures S3 and S4 shows the relationship between the posterior credible interval (PCI) for the mean and standard deviation parameters, respectively, and the amount of testing error. These results show similar levels of bias for the parameters regardless of testing error, indicating that the hindcasting framework is robust against even high levels of noise. However, the width of the PCI did increase with increasing levels of noise.

it should be pointed out that the ability of the MCMC sampler took longer to converge was when using the lowest test variability of 1.1 - likely because this assumption of an unrealistically accurate test mean that the peaks of the posterior distribution is much sharper, making it more difficult to traverse.


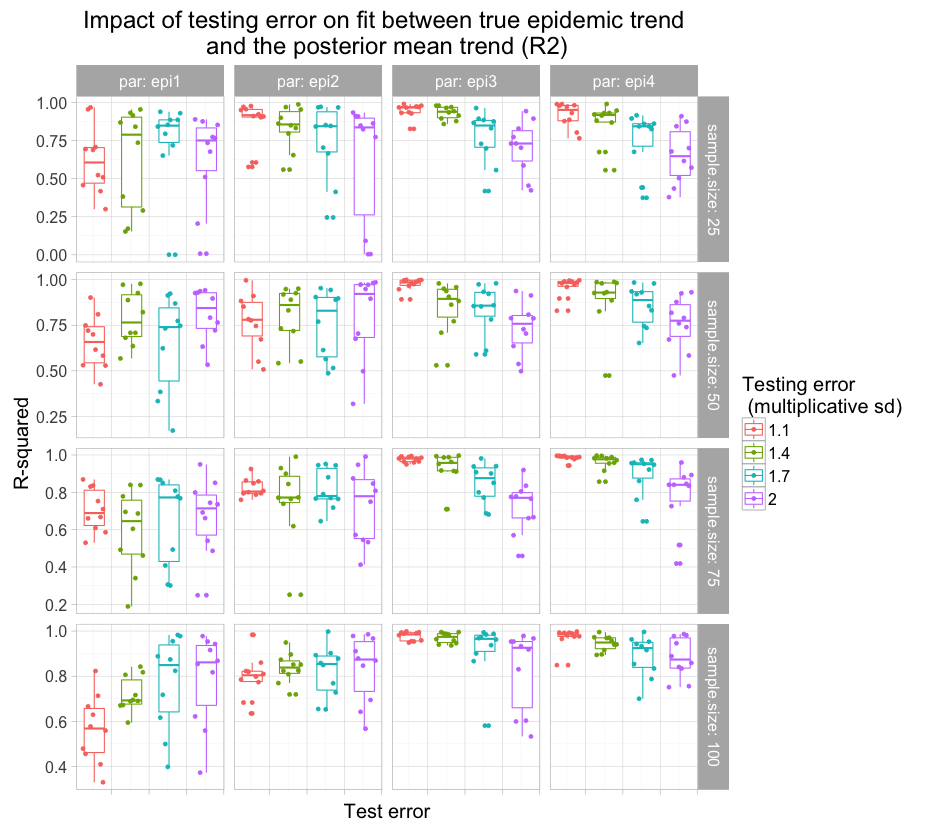
 **Figure S1: Impact of testing error on fit between true epidemic trend and the posterior mean trend, as measured with** R^2^**. Each dot represents the result of applying the hindcasting framework to a unique data set, generated with the indicated combination of sample size, testing error, and parameterization of epidemic trend. There were 10 datasets generated for each distinct combination of parameters – the distribution of these results are also summarized using boxplots, where the boxes indicate the 25th, 50th, and 75^th^ quantile. The level of testing error is indicated by position on the x-axis and by the colour of points and boxplots.**


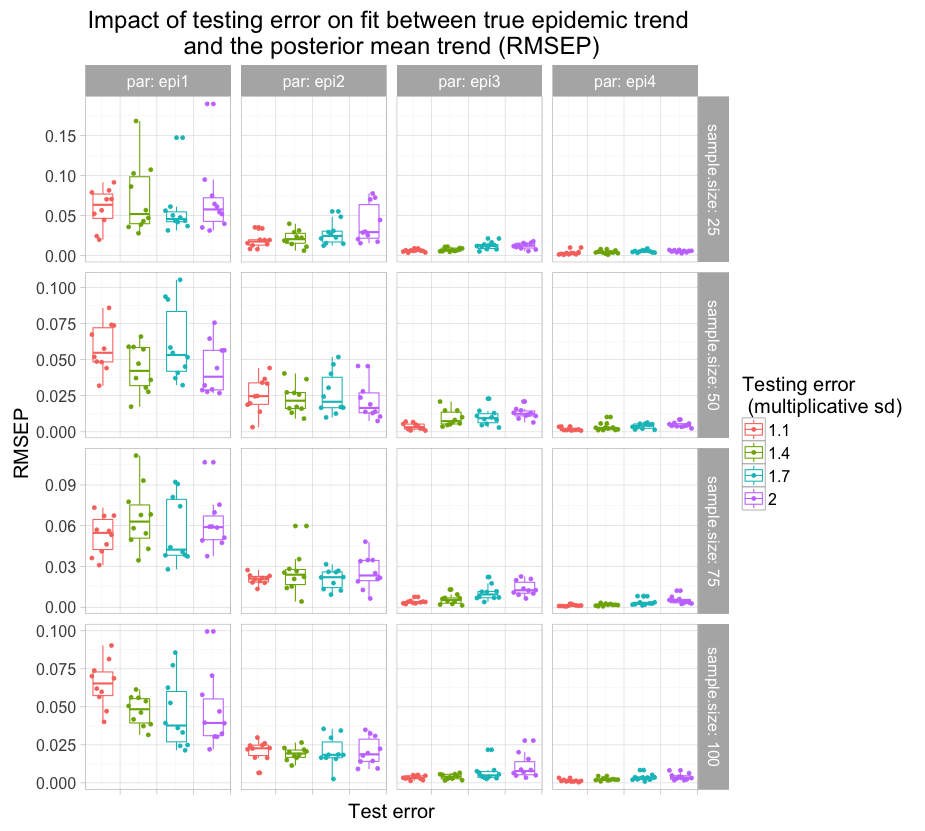
 **Figure S2: Impact of testing error on fit between true epidemic trend and the posterior mean trend, as measured with RMSEP. Each dot represents the result of applying the hindcasting framework to a unique data set, generated with the indicated combination of sample size, testing error, and parameterization of epidemic trend. There were 10 datasets generated for each distinct combination of parameters – the distribution of these results are also summarized using boxplots, where the boxes indicate the 25th, 50th, and 75^th^ quantile. The level of testing error is indicated by position on the x-axis and by the colour of points and boxplots.**
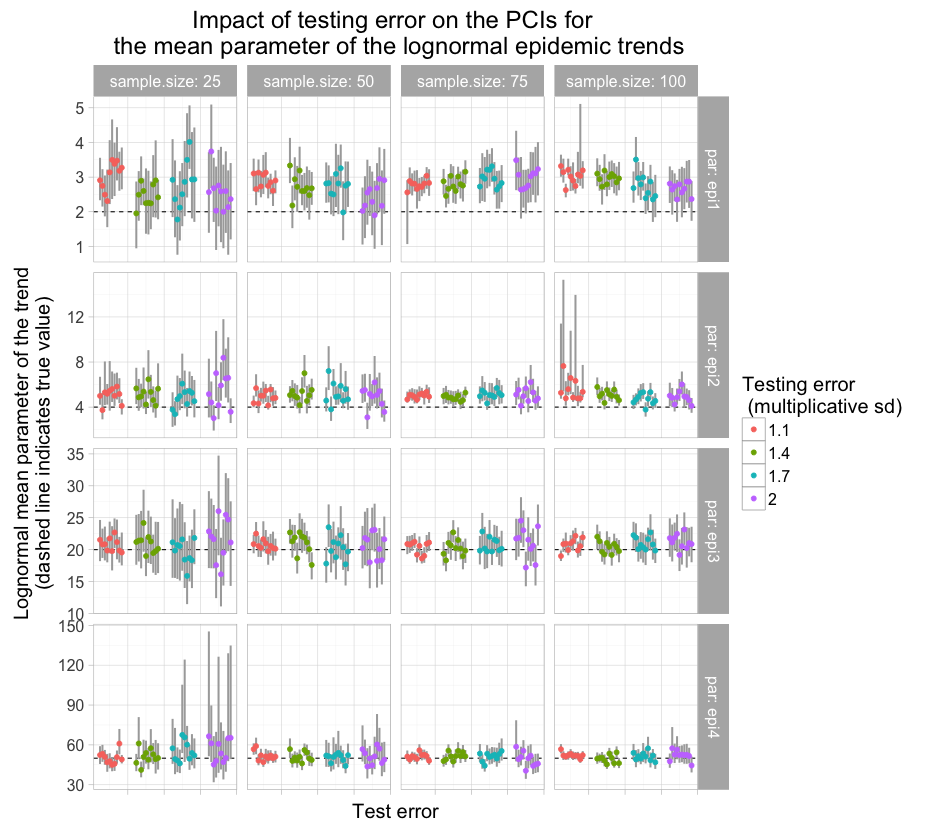
 **Figure S3: Impact of testing error on the 95% posterior credible interval (PCI) of the mean parameter of the epidemic trend. Each dot represents the result of applying the hindcasting framework to a unique data set, generated with the indicated combination of sample size, testing error, and parameterization of epidemic trend. The grey bars indicate the width of the PCIs. Horizontal dashed lines indicate the true parameter value. There were 10 datasets generated for each distinct combination of parameters – the distribution of these results are also summarized using boxplots, where the boxes indicate the 25th, 50th, and 75^th^ quantile. The level of testing error is indicated by position on the x-axis and by the colour of the points.**


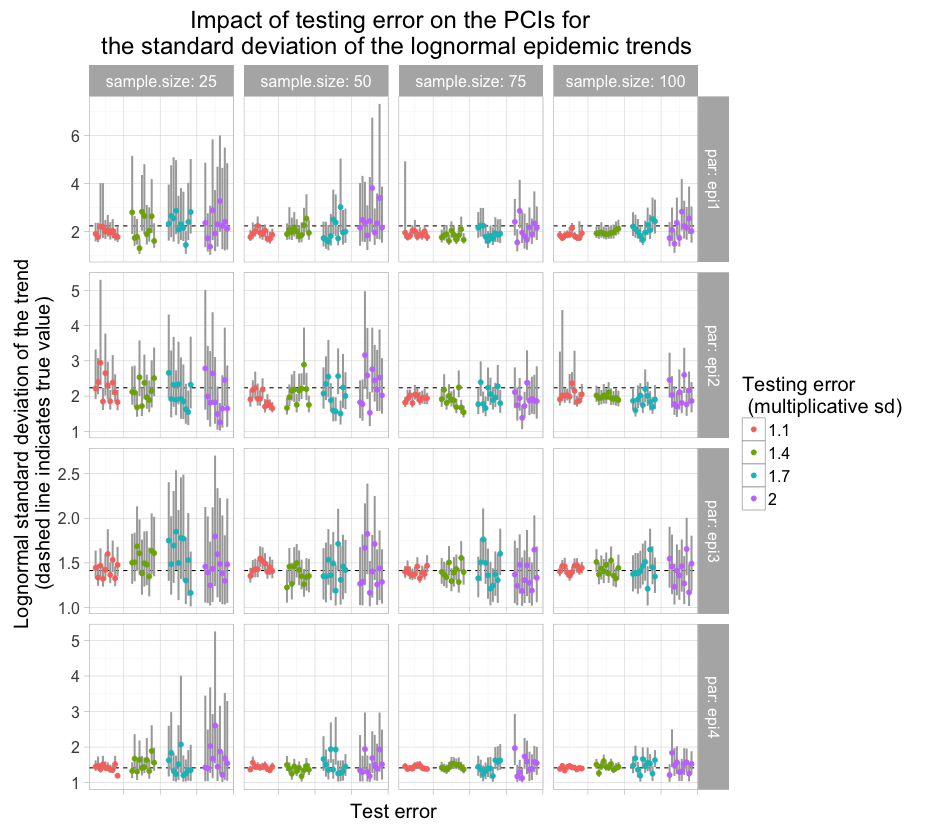
 **Figure S4: Impact of testing error on the 95% PCI of the standard deviation parameter of the epidemic trend. Each dot represents the result of applying the hindcasting framework to a unique data set, generated with the indicated combination of sample size, testing error, and parameterization of epidemic trend. The grey bars indicate the width of the PCIs. Horizontal dashed lines indicate the true parameter value. There were 10 datasets generated for each distinct combination of parameters – the distribution of these results are also summarized using boxplots, where the boxes indicate the 25th, 50th, and 75^th^ quantile. The level of testing error is indicated by position on the x-axis and by the colour of the points.**

**Violating the assumption of independent measurement errors**

In the current implementation of the hindcasting framework, it is assumed that the testing errors of the two diagnostic tests are independent, conditional on the expected mean as given by the test kinetics. While it is likely that much of the the overall dependence between the tests is captured by explicitly modelling the kinetics, it may be that there is additional residual dependence between the tests. We therefore ran a set of simulations that violated this assumption by varying the correlation of the testing errors from 0 to 1, in steps of 0.25. Figures S5-S6 shows the relationship between fit of the hindcasted trends and level of correlation. Figures S7-S8 shows the relationship between the level of correlation and the width and bias of the PCIs of the epidemic parameters. We could not identify any detrimental effect on fit as measured by R^2^ or RMSEP, regardless of the level of correlation between the two tests. We also failed to identify any effect on the bias and width of the PCIs.


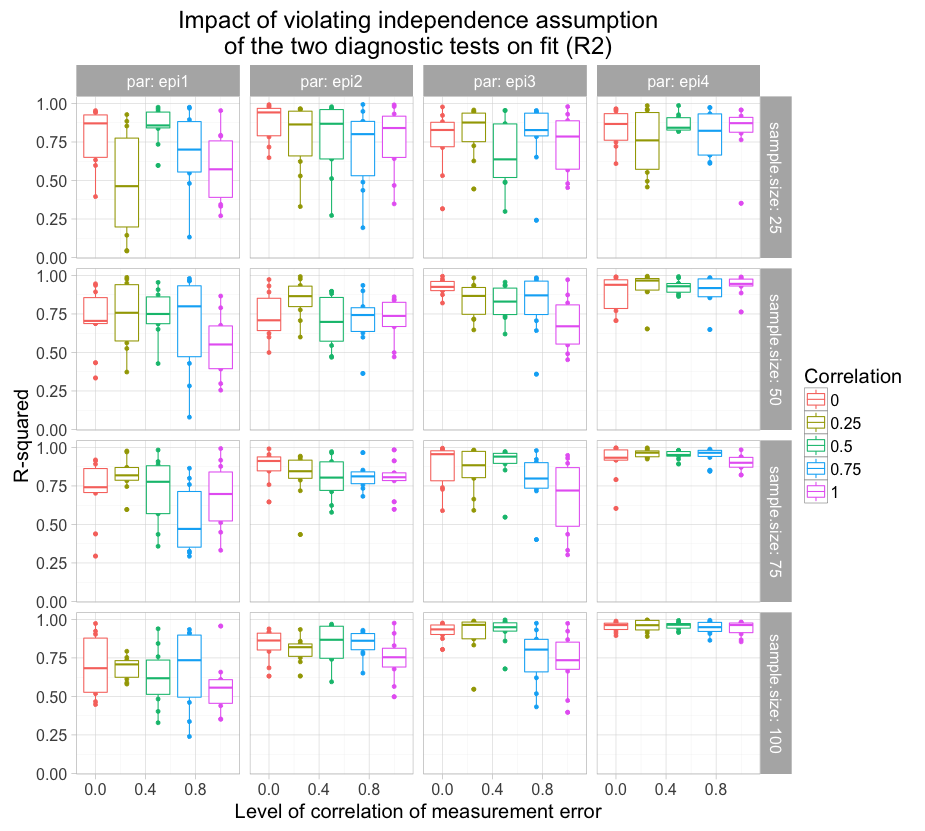
 **Figure S5: Impact of increasingly correlated diagnostic test results on the fit between true epidemic trend and the posterior mean trend, as measured with** R^2^**. Each dot represents the result of applying the hindcasting framework to a unique data set, generated with the indicated combination of sample size, test correlation, and parameterization of epidemic trend. There were 10 datasets generated for each distinct combination of parameters – the distribution of these results are also summarized using boxplots, where the boxes indicate the 25th, 50th, and 75^th^ quantile. The level of correlation between the two diagnostic tests is indicated by position on the x-axis and by the colour of points and boxplots.**


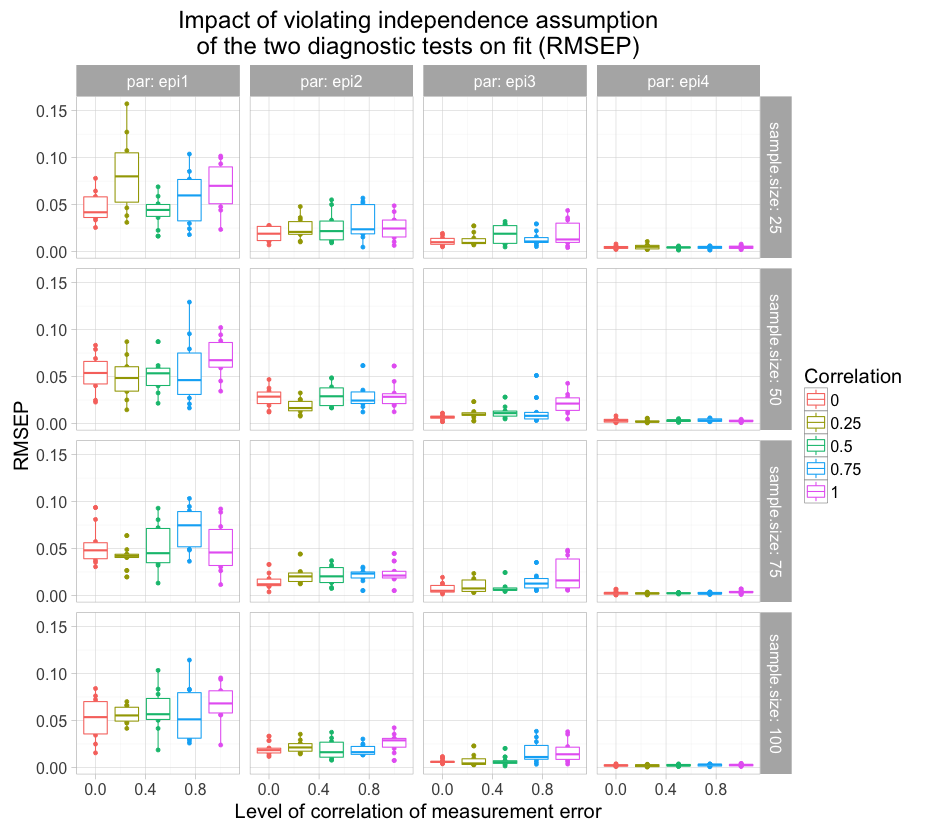
 **Figure S6: Impact of increasingly correlated diagnostic test results on the fit between true epidemic trend and the posterior mean trend, as measured with RMSEP. Each dot represents the result of applying the hindcasting framework to a unique data set, generated with the indicated combination of sample size, test correlation, and parameterization of epidemic trend. There were 10 datasets generated for each distinct combination of parameters – the distribution of these results are also summarized using boxplots, where the boxes indicate the 25th, 50th, and 75^th^ quantile. The level of correlation between the two diagnostic tests is indicated by position on the x-axis and by the colour of points and boxplots.**
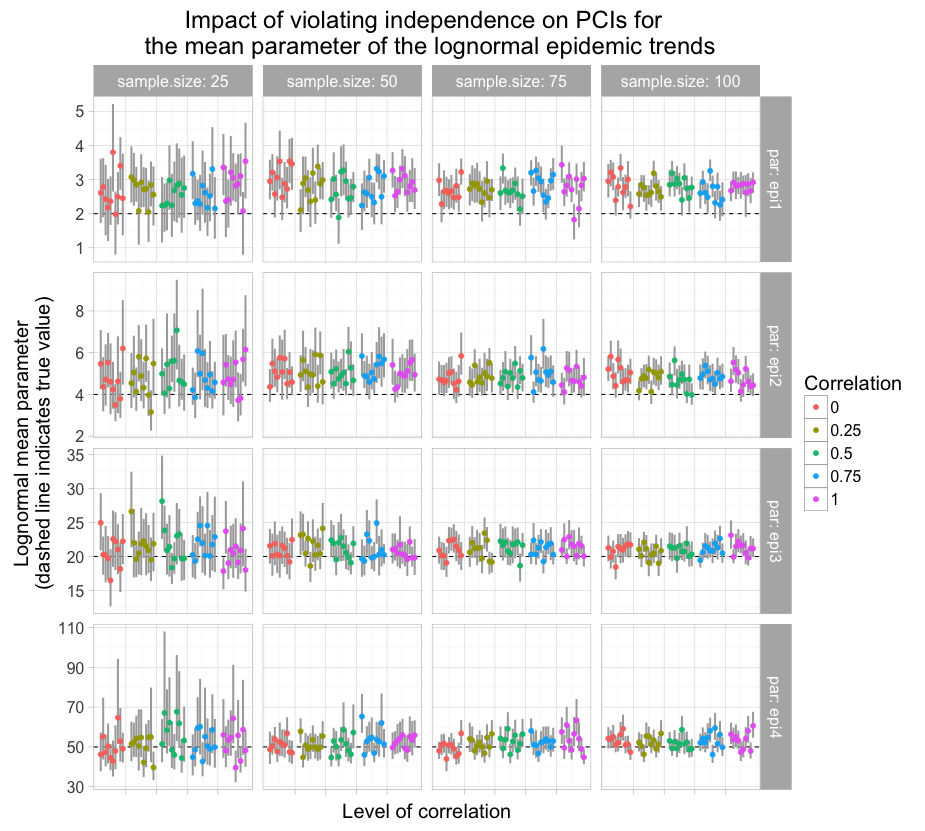
 **Figure S7: Impact of increasing correlation between the two diagnostic tests on the 95% PCI of the mean parameter of the epidemic trend. Each dot represents the result of applying the hindcasting framework to a unique data set, generated with the indicated combination of sample size, correlation between the two tests, and parameterization of epidemic trend. The grey bars indicate the width of the PCIs. Horizontal dashed lines indicate the true parameter value. There were 10 datasets generated for each distinct combination of parameters – the distribution of these results are also summarized using boxplots, where the boxes indicate the 25th, 50th, and 75^th^ quantile. The level of correlation is indicated by position on the x-axis and by the colour of the points.**


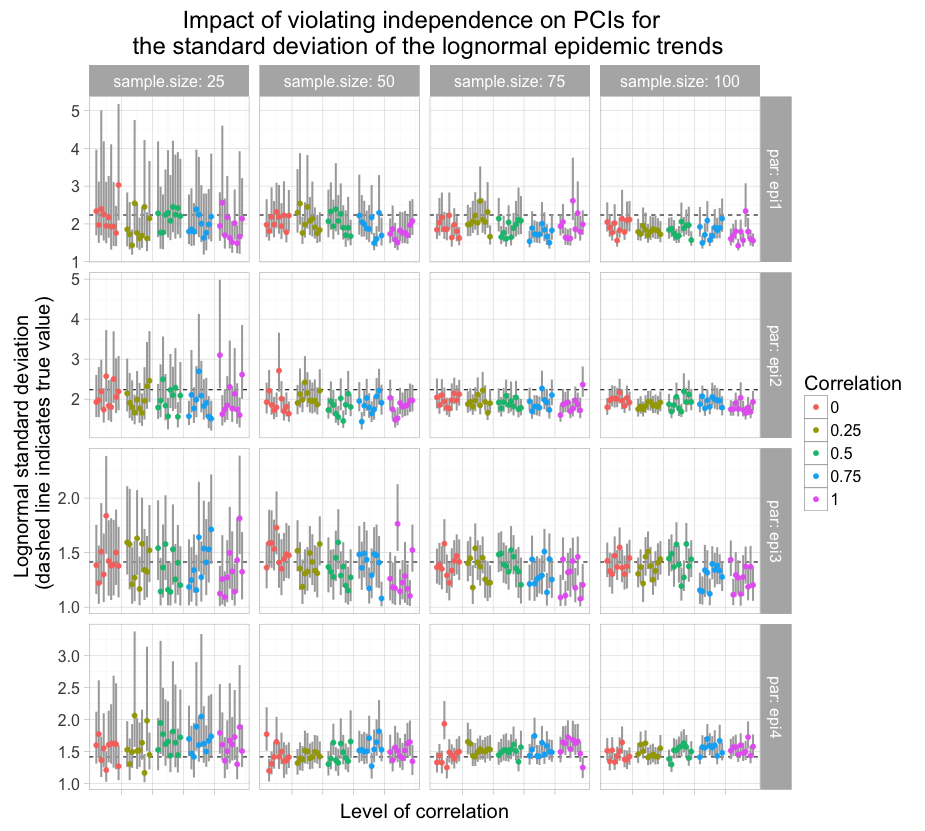
 **Figure S8: Impact of increasing correlation between the two diagnostic tests on the 95% PCI of the standard deviation parameter of the epidemic trend. Each dot represents the result of applying the hindcasting framework to a unique data set, generated with the indicated combination of sample size, correlation between the two tests, and parameterization of epidemic trend. The grey bars indicate the width of the PCIs. Horizontal dashed lines indicate the true parameter value. There were 10 datasets generated for each distinct combination of parameters – the distribution of these results are also summarized using boxplots, where the boxes indicate the 25th, 50th, and 75^th^ quantile. The level of correlation is indicated by position on the x-axis and by the colour of the points.**

**Implementation and results of running simulations with two diagnostic tests with increasingly similar test kinetics**

To evaluate the effect of the level of similarity between test kinetics, we created increasingly similar kinetic curves by taking a weighted average of the two kinetic curves $T1(t)$ and $T2(t)$ at each time point to generate a new diagnostic test kinetic

$T_{W}\left( t \right)=w*T1\left( t \right)+\left( 1-w \right)*T2(t)$.

We used different values of the weight $w$ to generate a family of test kinetics, where $w=0$ gives back test T2, and $w=1$ gives T1. For the purposes of this simulation, we started with the original paired test kinetics of BTV and Pertussis as described in the main paper. We then kept one of the tests constant, and used this as T1, and substituted the weighted $T_{W}\left( t \right)$ for the other test. The family of increasingly similar tests can be seen in figure S9. These test kinetics were then used in the hindcasting procedure to fit the four parameterizations of lognormal epidemics described above, assuming a sample size of 50, running the MCMC for 10000 iterations (including a 1000 iteration burn-in), followed by running it for 5000 iterations and taking a sample every 10^th^ iteration. This thinning was done to ensure sufficient convergence to elicit patterns despite the increasingly similar test kinetics. We generated 5 unique datasets for each combination of test kinetics and epidemic parameterization, and calculated the R^2^, RMSEP, and Gelman’s R of the resulting posterior samples. The results can be seen in figure S10- S12. We used Gelman’s R<1.1 as a criteria for convergence, and excluded non-converged iterations from the plots of R^2^ and RMSEP.

Figures S11 and S12 indicate that that for the BTV scenarios, the level of similarity of the tests did not seem to noticeably affect the accuracy (as measured with R^2^/RMSEP) of the estimated trends, while for the Pertussis scenarios when recovering Epi3 and Epi4 scenarios, performance degraded gradually, with a very low R^2^ when using two identical tests.

Conversely, the behaviour in terms of MCMC convergence was reversed. When using BTV test kinetics, the MCMC sampler converged well for the unmodified test configuration (as measured using Gelmans R statistic), but the convergence behaviour then degraded as the tests became increasingly similar. It completely failed to converge in the limit when the two tests were identical (either identical NA tests, or identical antibody based tests).  For the Pertussis scenarios the sampler converged for all combinations of diagnostic tests, no matter how similar.

That the BTV and Pertussis scenarios react so different could possibly be explained by a) the monotonic behaviour of the bacterial load of the Pertussis test compared to the sharp peak of the viral load of the BTV test, and b) the different peak times of the antibody curves which mean that BTV has a more diffuse likelihood function early on following exposure than does Pertussis.


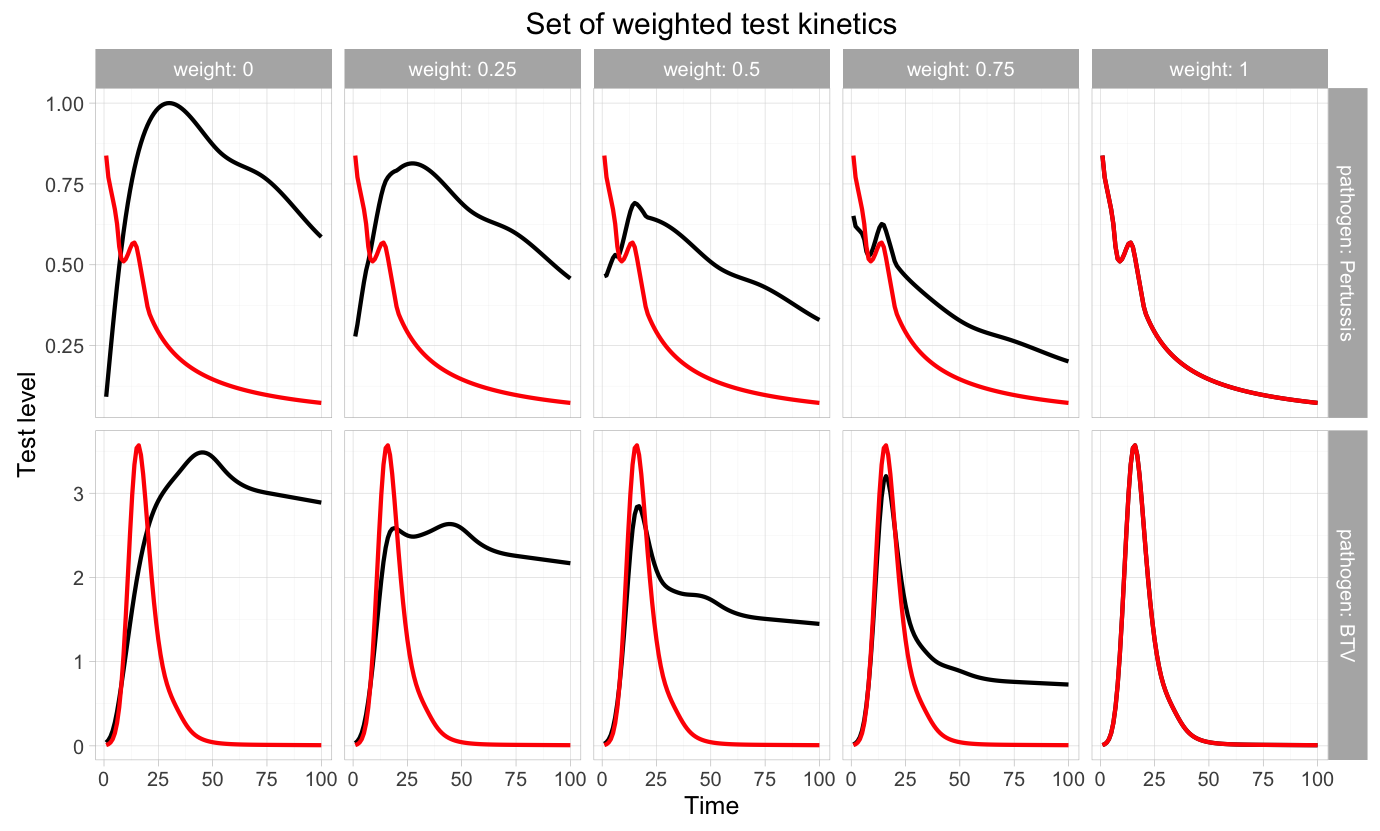
 **Figure S9: Test kinetics modified to become increasingly similar. Red lines indicate the fixed reference test. Black lines indicate the modified kinetics. The weight parameter indicates the relative weight of the reference test in the weighted average. The first column with weight=0 thus show the two unmodified kinetics.**


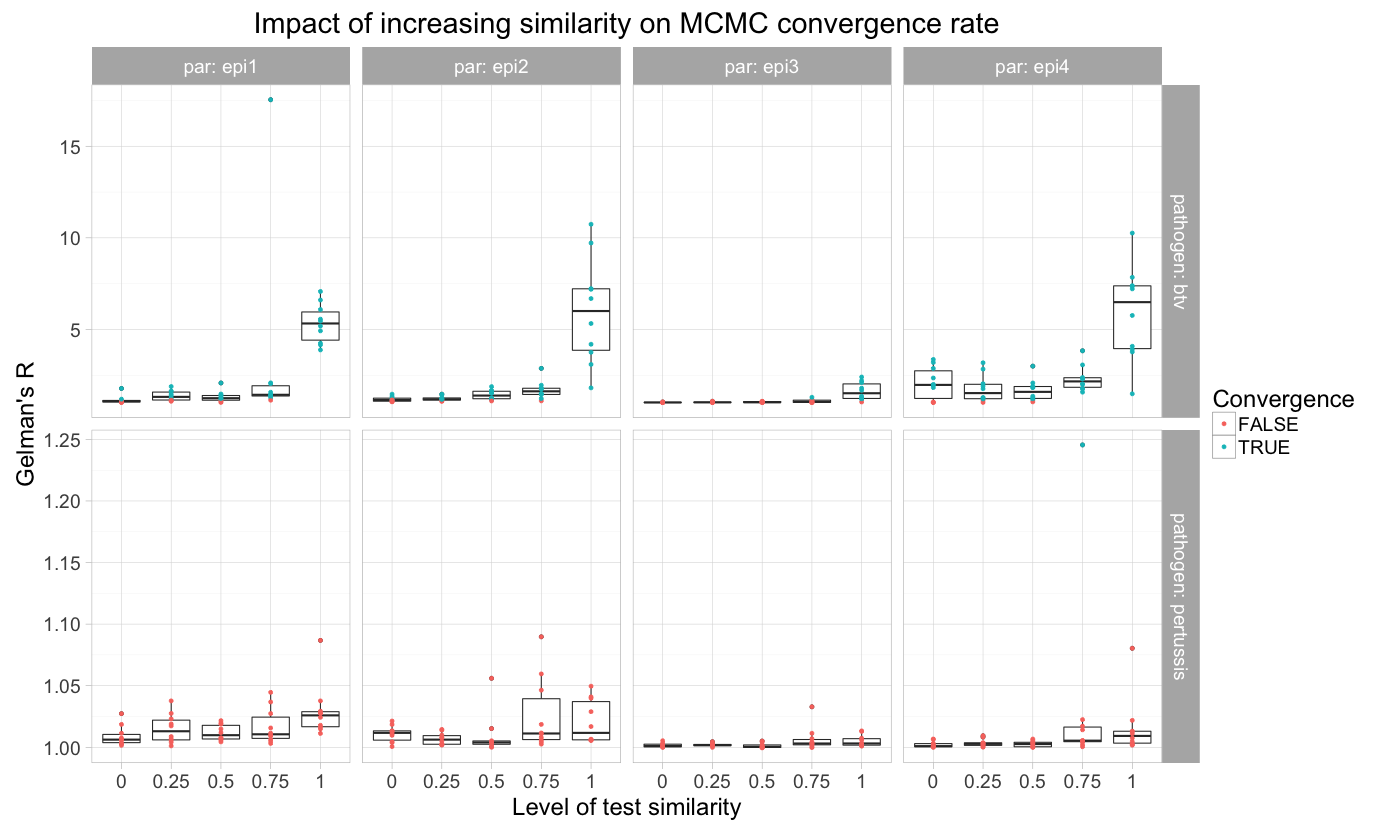
 **Figure S10: Convergence of the MCMC chain versus test similarity, as measured with Gelman’s R. Each boxplot summarises the results of fitting the framework to ten different datasets. Gelman’s R was calculated after a burn-in period of 10,000 iterations, and subsequent sampling of every 10^th^ iteration for 5000 steps, for a total of 500 posterior samples. Rows indicate the type of pathogen used, columns indicate the parameterization of the epidemic trend. The colour is red if Gelman’s R is under 1.1 for the posterior samples (indicating that convergence has been reached), and blue otherwise.**


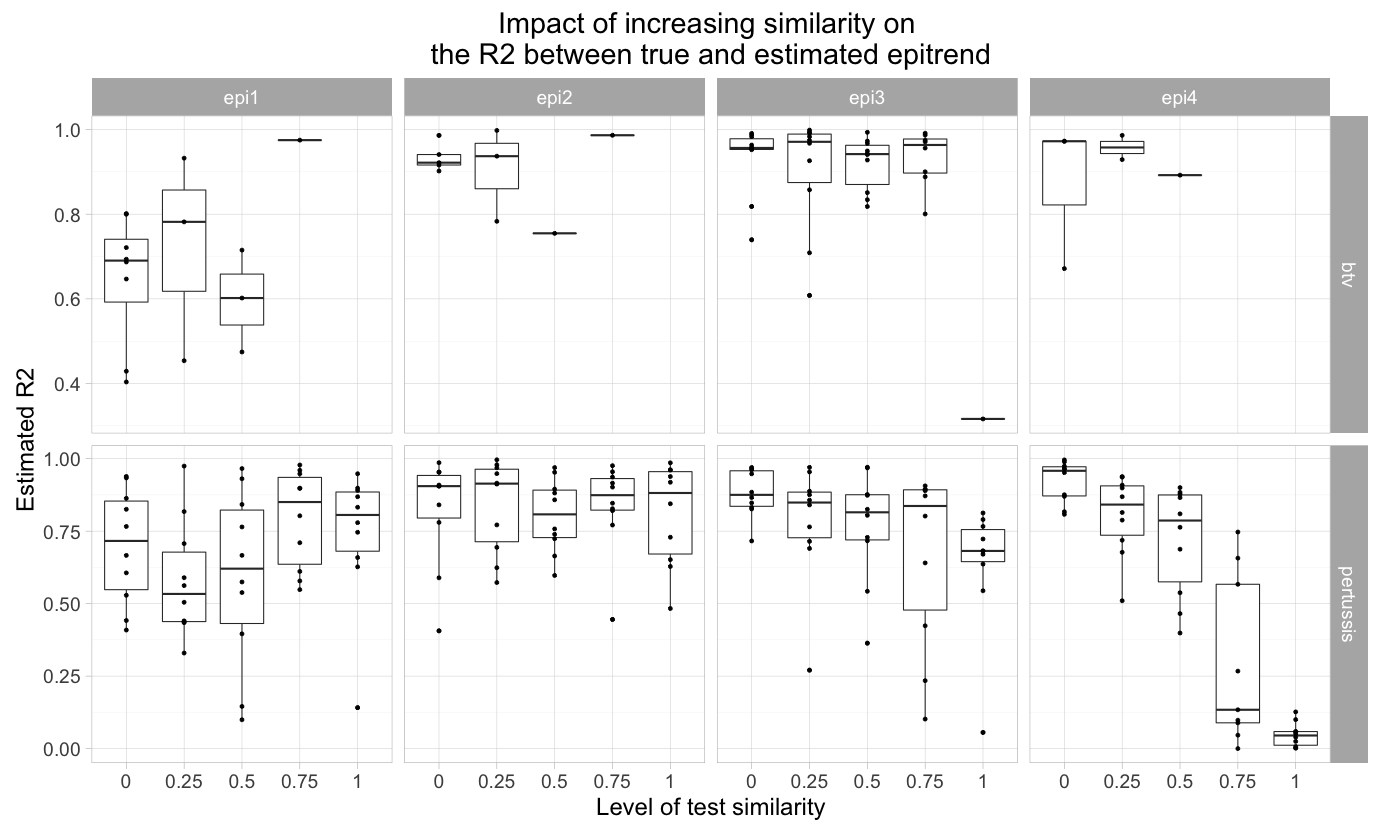
 **Figure S11: Fit of the estimated posterior trend as compared to the true trend, measure with** R^2^**. Only results that had reached convergence as indicated by Gelman’s R is 1.1 is included. Each box summarises the results of simulations with the same set of parameter values for the scenario.**


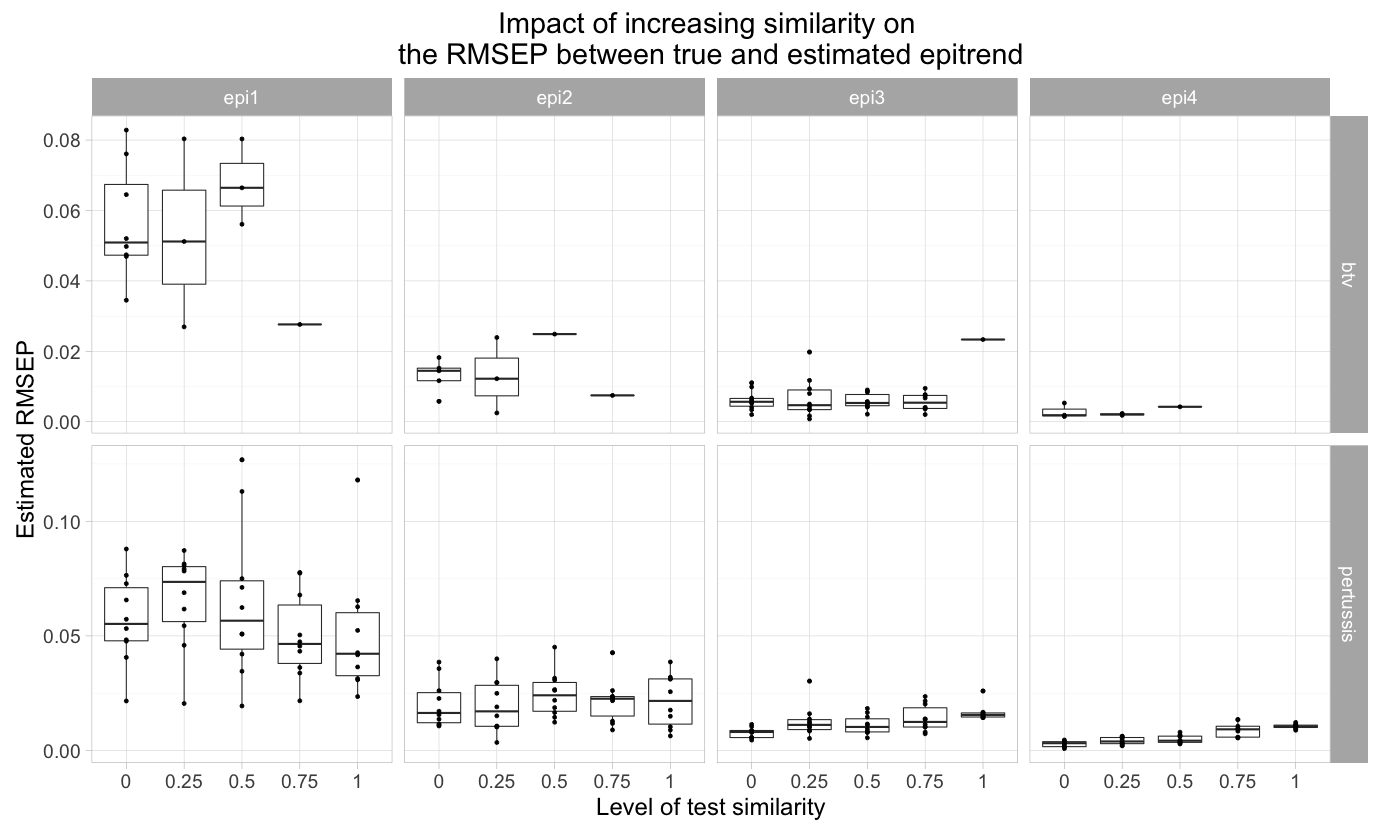
 **Figure S12: Fit of the estimated posterior trend as compared to the true trend, measure with RMSEP. Only results that had reached convergence as indicated by Gelman’s R is 1.1 is included. Each box summarises the results of simulations with the same set of parameter values for the scenario.**

**Sensitivity to values of priors**

In order to ensure the robustness of our hindcasting framework, we conducted a sensitivity analysis of the choice of priors. We used a lognormal prior distribution for the mean parameter of the epidemic trend distribution. Figures S13 show the effect of different choices of the mean and standard deviation of the prior distribution on the estimate of the mean of the epidemic trend. As can be seen, there was no difference in the posterior estimate over a very wide range of prior distribution values. A similar (non)pattern was seen for the posterior estimate of the standard deviation of the epidemic trend.


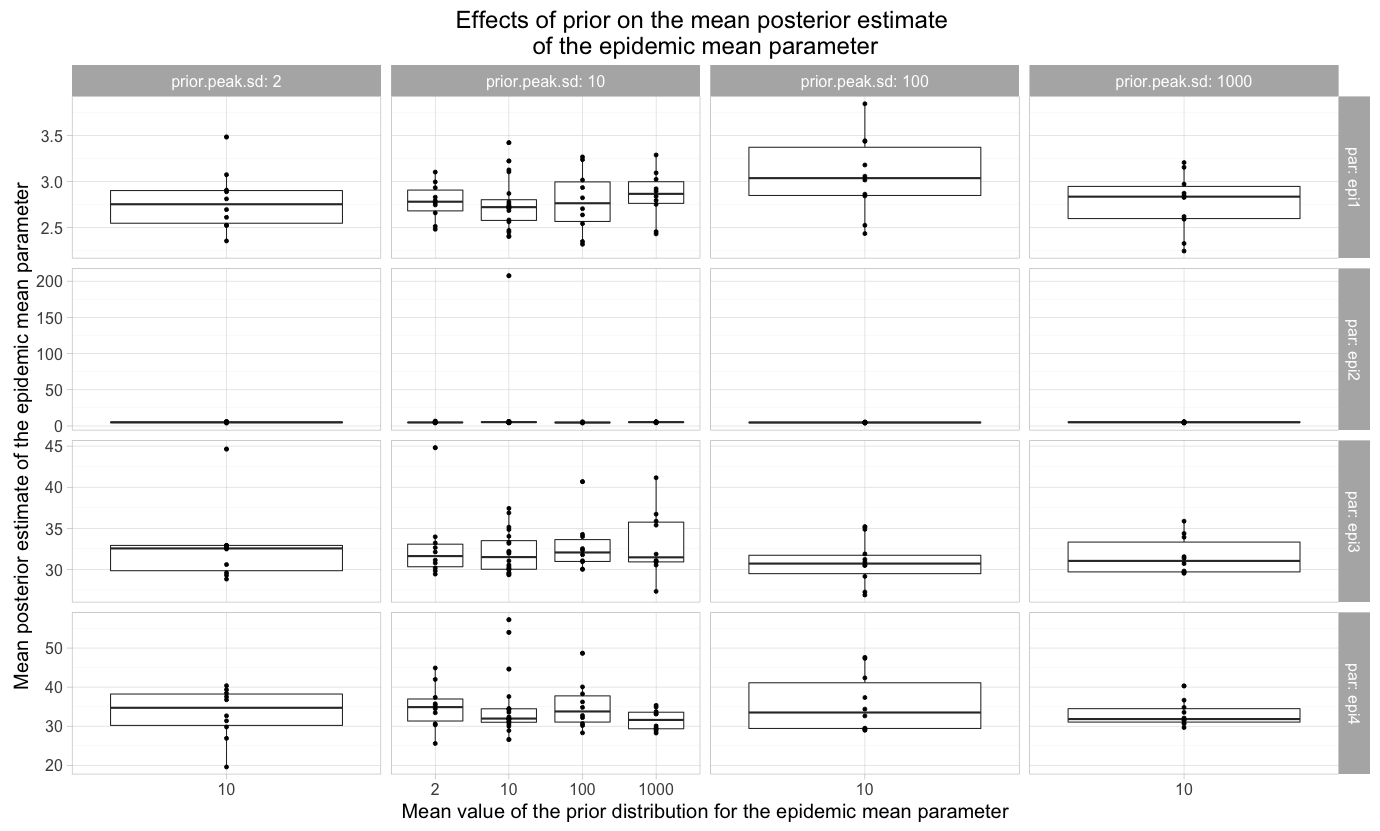
 **Figure S13: Relationship between the choice of priors and the posterior estimate of the mean of the epidemic trend. Columns indicate different values of the standard deviation parameter of the prior; columns indicate the four different types of parameterizations of the epidemic trend. The x-axis indicates the different values of the mean parameter of the prior distribution, and the y-axis indicates the mean posterior estimate. Each boxplot summarise the result of the hindcasting framework applied to 10 different datasets, generated using 50 samples each, and a test error of 1.5.**

**Evaluation of convergence**

When evaluating convergence of the MCMC runs used to estimate the parameters of the epidemic trends of outbreaks (see the results section in main body of the paper), the Gelman-Rubin (GR) statistic[2.1] was used as a first indicator. This widely used statistic [2.2] measures the ratio of the within-chain variability to the between-chain variability e.g. for multiple chains started from different initial conditions.

The convergence behaviour was quantified of the MCMC algorithm for all runs used, across all different scenarios, type of disease diagnostic, the number of individuals tested, etc. (see main text). For the main scenarios based on a synthetic lognormal epidemic trend, the MCMC samples were generated using a 9500 iteration burn in, and subsequent capturing every third of the following 1500 iterations, for a total of 500 samples from the posterior. In each case 5 chains were run in parallel to ensure sufficient information on between-chain variability

For the similarity scenarios, this was increased to a 10000 burn-in, followed by sampling every 5^th^ of 5000 iterations. For the the case studies, this was further increased to a 10000 burn-in, followed by sampling every 10^th^ of 10000 iterations.

Fig S14 show examples of traceplots from the case studies, which were the scenarios with the slowest convergence behavior (likely because they didn’t have as clear a peak as the synthetic scenarios). These were fitted to diagnostic test results from 100 individuals. The patterns of the bluetongue traceplots indicate that that chains are mixing slowly, which explains the low values of the GR statistic obtained. Fig S15 shows the estimated epidemic trends using mean parameter values from the final 5000 iterations of the MCMC algorithm, with separate trends for each of the five chains. These plots indicate that despite the slow mixing, the practical difference between chains was miniscule.

**
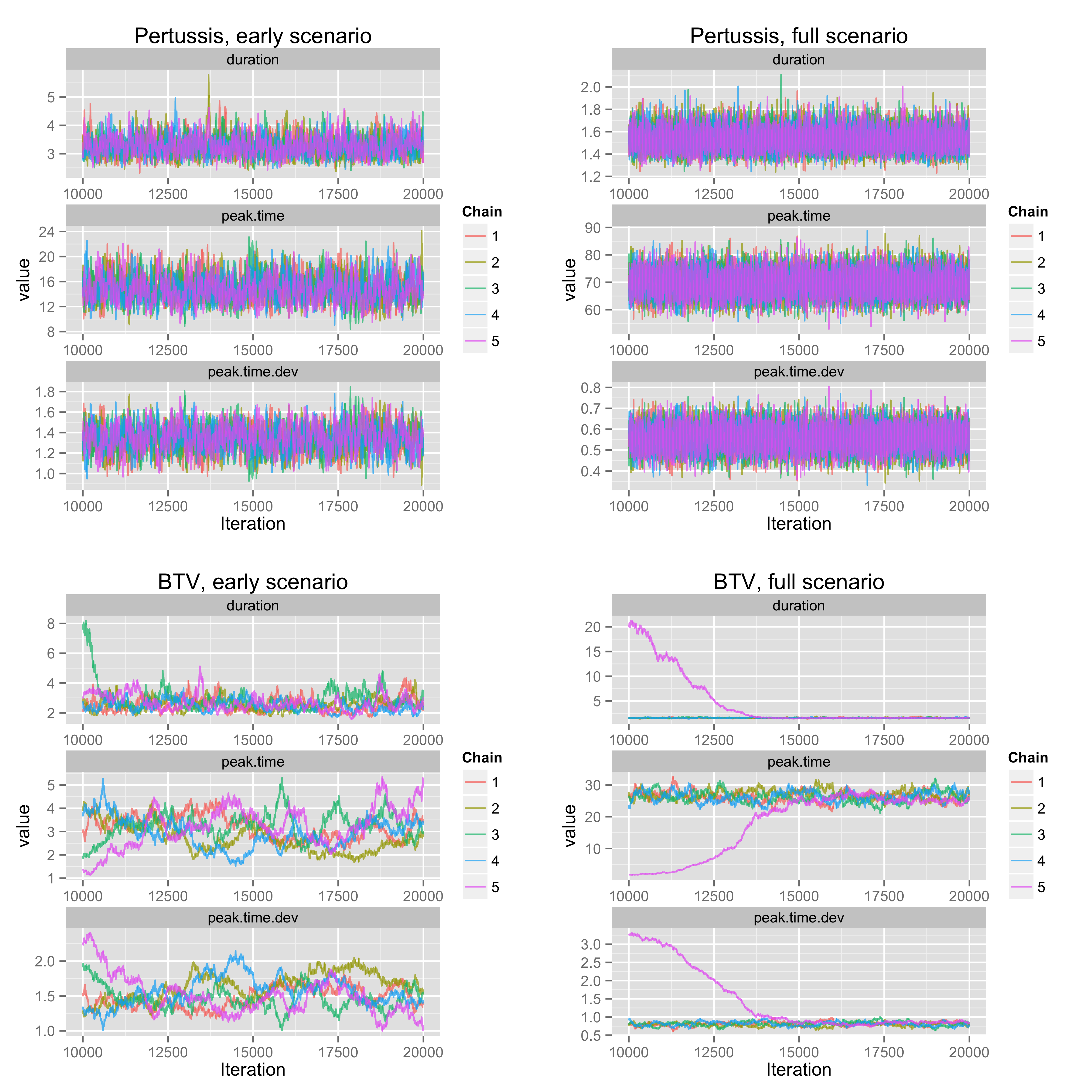
**

**Figure S14: Examples of MCMC traceplots, generated by fitting the hindcasting model to scenarios assuming that disease diagnostics were collected from 100 individuals. Each colour indicates a different chain (i.e. started with different initial conditions). “Peak.time” and “duration” are the parameters for the mean and variance of the lognormal distribution describing the epidemic trends. “Peak.time.dev” is the variance of the prior of peak.time.**


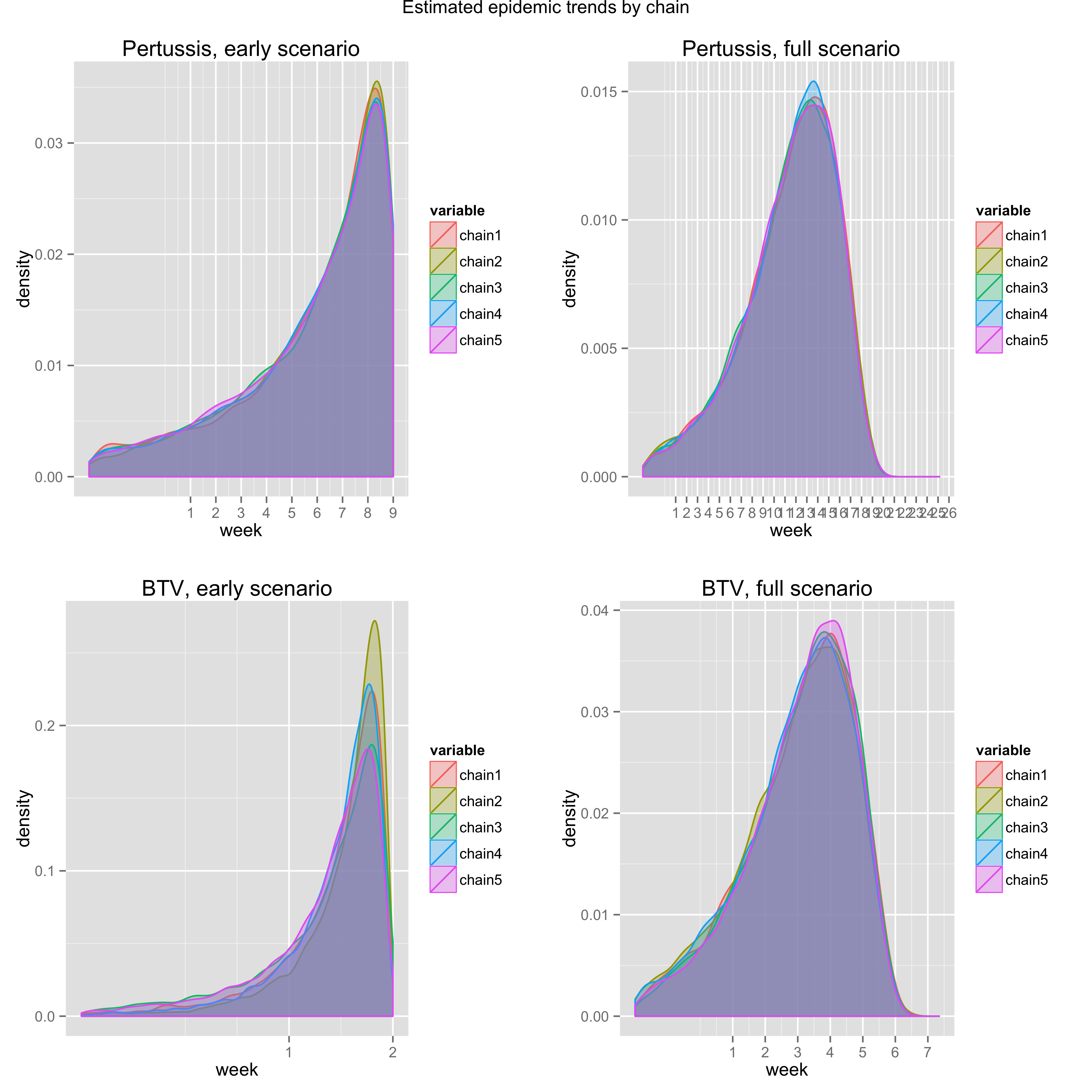


Figure S15: Estimated epidemic trends by chain. The parameters of the lognormal distribution describing the epidemic trend was calculated from the mean of the last 5000 samples of “peak.time” and “duration” in the traceplots in Fig S14, above.

**Generating simulated data**

The BTV and *B. pertussis* data sets we applied the hindcasting inference framework to were simulated in R using the following procedure:

1. Choose an observation time$T$ after the first recorded case of the epidemic we base our simulated data on.
2. Record the observed exposure times $\boldsymbol{E}=\{e_{i}\}$ for all cases that had occurred in the real epidemic up until the time of observation $T$ and evaluate the duration of infection/exposure for each individual $\{d_{i}=T-e_{i}\}$ at time $T$.
3. Use the deterministic function $\boldsymbol{l(}d_{\boldsymbol{i}}\boldsymbol{)}$ based on the interpolated test trajectory to assign mean test values for each case given the durations of infection
4. Set the variance $\Sigma^{2}$ of the log-normal distribution to correspond to published test variability.
5. Generate test results from the log normal distribution with $\Sigma^{2}$ variance and mean test values generated in steps 3 and 4.

Test scores corresponding to viral load and antibody response are then simulated using the deterministic function $\boldsymbol{L}=\left\{ \boldsymbol{l}\left( d_{i} \right) \right\}\equiv\{vl\left( d_{i} \right),ab\left( d_{i} \right)\}$ described in the methods section. Note that the infection times are not subsequently used in the inference procedure, but they do provide an opportunity to assess the inferences obtained.

The lognormal variation around this curve was set to be 57% following published data on *B. pertussis* antibody variability [35]. DNA measurements and antibody measurements were assumed to have the same level of noise.

Based on published data [2.3] on the variability of RT-PCR as applied to BTV, we set the log-normal variability to 27%. For simplicity, we assume equal variability for antibody measurements.

**An alternative formulation of the lognormal distribution to allow the MCMC sampler to efficiently sample multimodal likelihood surfaces**

As shown in figure 3 in the main text, the likelihood for times since exposure is often multimodal. Combining multiple tests can reduce this problem, but if the MCMC sampler is initiated in the wrong region, convergence can be an issue where the chain becomes ‘stuck’ around one mode with an extremely small probability of jumping to an alternative mode. By allowing the MCMC sampler to jump between disjoint regions of exposure times in one step, the different modes are no longer isolated from each other. The following describes how to implement such a solution in the case of the lognormal distribution used in the paper.

Asssume that times of exposure $\boldsymbol{e}_{i}$ are lognormally distributed, $e_{i}\sim logN(\mu, \sigma)$ For the standard lognormal parametrization, the density for a particular time of exposure $\boldsymbol{e}$ given mean $\mu$ and variance $\sigma$ is given by

$$p\left( \boldsymbol{e} | \mu,\sigma\right)=lN\left( \boldsymbol{e}|\mu,\sigma\right)=N\left( \log\left( \boldsymbol{e} \right) | \mu,\sigma\right)=\phi(\frac{\log\left( \boldsymbol{e} \right)-\mu}{\sigma})$$

, where $\phi\left( x \right)=N\left( x \right|\mu=0, \sigma=1)$.

Tautologically, we can rewrite $\boldsymbol{e}$ as

$$\boldsymbol{e}=e^{\log\left( e \right)}=e^{\mu+\log\left( \boldsymbol{e} \right)-\mu}=e^{\mu+[(log \left( \boldsymbol{e} \right)-\mu)/\sigma]*\sigma}= e^{\mu+\Delta*S*\sigma}$$

where $S\in\left\{ -1,1 \right\}$, and $\Delta=\left| \frac{\log\left( \boldsymbol{e} \right)-\mu}{\sigma} \right|\geq0$.

Now, instead of assuming that $\boldsymbol{e}$ is coming from a lognormal distribution, we can

assume that S has a discrete probability function,

$$a) p\left( S=1 \right)=p\left( S= -1 \right)=0.5$$

, and that $\Delta$ has a folded standard normal distribution with a probability density function given by

$$b) p\left( \Delta, \Delta>0 \right)=\phi\left( \Delta\right)+\phi\left( -\Delta\right)=2\phi\left( \Delta\right)$$

In this way, $\Delta$ can be interpreted as how far $\boldsymbol{e}$ is away from the mean of the lognormal distribution, measured in the number of standard deviations, and S indicates whether it is in the upper or lower quantile. Note that for fixed $\mu$and $\sigma$, each exposure time $\boldsymbol{e}$ can be written uniquely as a combination of $\Delta$ and $S$.

Using this formulation, the probability for **e** can be written as a product of $\Delta$ and $\sigma$:

$$p\left( \boldsymbol{e}|\mu,\sigma\right)=p\left( \Delta,S|\mu,\sigma\right)=p\left( \Delta\right)*p\left( S \right)$$

From a) and b), we get that

$$p\left( \Delta\right)*p\left( S \right)= 2*\phi\left( \Delta\right)*0.5=\phi(\Delta)=\phi(|\frac{\log\left( \boldsymbol{e} \right)-\mu}{\sigma}|)$$

Thus, (since the normal distribution is symmetric) this new formulation results in the same probability distribution for T as the lognormal distribution, and thus an equal contribution to the data likelihood. For the bluetongue and whooping cough examples, the full posterior likelihood is written as

$$L\left( \boldsymbol{E},\theta| \boldsymbol{Y},\boldsymbol{T} \right)\boldsymbol{\sim}\left( \prod_{\forall i} \boldsymbol{( l}\mathcal{N(}\boldsymbol{Y|L}\left( \boldsymbol{T}-e_{i} \right),\Sigma^{2}))\boldsymbol{l}\mathcal{N}(e_{i}\left| \mu, \sigma\right) \right)Prior\left( \mu\right)Prior(\sigma)$$

Using the new formulation, this becomes

$$p\left( \boldsymbol{E},\theta| \boldsymbol{Y},\boldsymbol{T} \right)\boldsymbol{\sim}$$

$\left( \prod_{\forall i} \left( \boldsymbol{l}\mathcal{N}\left( \boldsymbol{L}\left( T-e^{\mu+\Delta_{i}*S_{i}*\sigma} \right),\Sigma^{2} \right) \right)\phi\left( \Delta_{i} \right)*2*p(S_{i}) \right)Prior\left( \mu\right)Prior(\sigma)$=

$$\left( \prod_{\forall i} \left( \boldsymbol{l}\mathcal{N}\left( \boldsymbol{L}\left( T-e^{\mu+\Delta_{i}*S_{i}*\sigma} \right),\Sigma^{2} \right) \right)\phi\left( \Delta_{i} \right) \right)Prior\left( \mu\right)Prior(\sigma)$$

In an MCMC setting, this formulation allows for generating a new proposal $T'$ by jumping from $\left( \Delta,S^{+} \right)\to(\Delta,S^{-})$, thus reducing the risk of getting stuck in local maximums of the likelihood. In effect, by decomposing $\boldsymbol{e}$ into two separate variables, we are adding an extra dimension that the MCMC sampler can jump through, bringing the separate modes closer together.

**Supplementary References**

2.1. Gelman A, Rubin DB (1992) Inference from Iterative Simulation Using Multiple Sequences. Stat Sci 7: 457–472.

2.2. Kathryn M, Carlin BP, Cowles MK (1996) Markov Chain Monte Carlo Convergence Diagnostics : A Comparative Review. J Am Stat Assoc 91: 883–904.

2.3. Chatzinasiou E, Dovas CI, Papanastassopoulou M, Georgiadis M, Psychas V, et al. (2010) Assessment of bluetongue viraemia in sheep by real-time PCR and correlation with viral infectivity. J Virol Methods 169: 305–315. Available: http://www.ncbi.nlm.nih.gov/pubmed/20691732. Accessed 11 December 2013.
